# Can adults learn L2 grammar after prolonged exposure under incidental conditions?

**DOI:** 10.1371/journal.pone.0288989

**Published:** 2023-07-26

**Authors:** Panagiotis Kenanidis, Ewa Dąbrowska, Miquel Llompart, Diana Pili-Moss

**Affiliations:** 1 Chair of Language and Cognition, Department of English and American Studies, Friedrich-Alexander-Universität Erlangen-Nürnberg, Erlangen, Germany; 2 Department of English Language and Linguistics, University of Birmingham, Birmingham, United Kingdom; 3 Department of Translation and Language Sciences, Universitat Pompeu Fabra, Barcelona, Spain; 4 Institute of English Studies, Faculty of Education, Leuphana Universität Lüneburg, Lüneburg, Germany; Potsdam University, GERMANY

## Abstract

While late second language (L2) learning is assumed to be largely explicit, there is evidence that adults are able to acquire grammar under incidental exposure conditions, and that the acquisition of this knowledge may be implicit in nature. Here, we revisit the question of whether adults can learn grammar incidentally and investigate whether word order and morphology are susceptible to incidental learning to the same degree. In experiment 1, adult English monolinguals were exposed to an artificial language (Kepidalo) that had case marking and variable word order: a canonical Subject-Object-Verb order and a non-canonical Object-Subject-Verb. In a five-session online study, participants received vocabulary training while being incidentally exposed to grammar, and completed a series of picture-selection and grammaticality judgment tasks assessing grammatical knowledge. Despite extensive exposure to input, and although performance on vocabulary increased significantly across sessions, learners’ grammatical comprehension showed little improvement over time, and this was limited to Subject-Object-Verb sentences only. Furthermore, participants were better at detecting word order than case marking violations in the grammaticality judgment tasks. Experiment 2 further increased the amount of incidental exposure whilst examining native speakers of German, which exhibits higher morphological richness. Testing was followed by a post-test metalinguistic awareness questionnaire. Although greater learning effects were observed, participants continued to have difficulties with case marking. The findings also demonstrated that language outcomes were modulated by learners’ level of metalinguistic awareness. Taken together, the results of the two experiments underscore adult learners’ difficulty with case marking and point towards the presence of a threshold in incidental L2 grammar learning, which appears to be tightly linked to prior first language experience. In addition, our findings continue to highlight the facilitative role of conscious awareness on L2 outcomes.

## Introduction

There is a general consensus that mastering a second language (L2) is a notoriously demanding task, particularly for adult learners, and, therefore, native-like attainment is very rarely achieved. Previous studies on L2 acquisition indicate that learning a language at a later stage in life is largely guided by explicit learning [1–3]. Consequently, studies comparing L2 learning under incidental and intentional conditions have demonstrated that the presentation of explicit information about the learning target leads to greater learning gains [4–6]. By contrast, first language (L1) acquisition is a process that is thought to occur mostly unconsciously and automatically (e.g., [7–10]).

Interestingly, however, a growing body of research on artificial language learning has provided evidence that participants can rapidly develop knowledge about different aspects of a novel (L2) grammar even under incidental conditions (e.g., [11–13]). Yet, a recurrent pattern in these studies is that participants typically achieve performance that is only slightly above chance and rarely exceeds 60% accuracy [14, 15]. These findings may be attributable to three different yet potentially overlapping factors. First, adults’ capacity to learn grammatical rules incidentally may, to a certain extent, be affected by maturational constraints, resulting in low learning rates [9, 16]. Second, language learning outcomes are frequently assessed after a limited amount of exposure to input, usually confined within a single session, which may be insufficient for learners to develop robust grammatical knowledge (for studies with an extensive language training regimen see [17, 18]). In fact, notwithstanding their differences, contemporary cognitive models of L2 acquisition (e.g., [2, 9, 19, 20]) converge in suggesting that repeated exposure to input and practice can lead to better language learning outcomes. Third, adult L2 attainment appears to be substantially affected by learners’ previous L1 experience [1, 21, 22]. Hence, given that in the majority of studies the target population consisted of native speakers of English [11–13], a fixed word order language, the extent to which previous learning outcomes can be attributed to limits in learning under incidental exposure conditions per se, or whether they are additionally modulated by previous L1 experience, is still far from clear.

The present study set out to revisit the question of whether adults can learn novel grammatical structures incidentally, while addressing the aforementioned gaps in the literature. To this end, we examined if word order and inflectional morphology are susceptible to learning under incidental conditions to the same extent, as well as the degree to which their learnability is affected by the amount of exposure to the novel language and the level of similarity between the L1 and the new target language. Furthermore, most studies have focused on testing the learning of one grammatical feature at a time (e.g., case-marking; [13, 17]; word order; [23–25]; noun-adjective agreement; [26]; verb morphology; [27]). Therefore, surprisingly, little is known about the order in which different aspects of grammar are acquired when adult learners are incidentally exposed to multiple grammatical features simultaneously. Comparing the simultaneous acquisition of various grammatical features can allow studying how participants weight novel linguistic cues, which one(s) they prioritize and how the development of one may influence the development of the other(s), thus providing important insights into the language acquisition process. Note that, in this paper, the terms acquisition and learning will be used interchangeably to refer to the process by which one learns language.

## Background

### Incidental and intentional L2 learning

As part of the effort to gain a better understanding of the fundamental cognitive mechanisms underlying L2 learning, researchers have explored how learners acquire linguistic knowledge under two different conditions: incidental and intentional exposure conditions. In this context, intentional exposure refers to the condition in which participants are given explicit information about the learning targets or are instructed to engage in deliberate hypothesis testing and memorization of rules [3, 28, 29]. Such conditions promote primarily the engagement of explicit learning processes, which are thought to contribute primarily to the development of explicit knowledge, often signified by learners’ ability to verbalize the acquired rules [29, 30]. In contrast, incidental exposure is operationalized as the situation where participants are not informed about the learning target and the subsequent test phase [29, 31]. To achieve this, a cover task is used which is intended to focus learners’ attention on another activity that requires processing the input for meaning, instead of overtly encouraging them to consciously focus on the linguistic structure. Learning under incidental conditions is considered to favor the involvement of implicit learning processes, which result in the acquisition of implicit knowledge [30].

Intentional and incidental conditions are usually conflated with the terms explicit and implicit, respectively, and they are occasionally used interchangeably. However, they are not identical. The former two terms are more appropriate for describing the experimental conditions researchers design to investigate the type of learning that is taking place, as well as the nature of the L2 knowledge participants develop. In contrast, the latter two refer to the internal learning process that is engaged while acquiring new knowledge. The distinction can account for previous findings showing that participants can engage both explicit and implicit learning processes and can acquire both explicit and implicit knowledge irrespective of the conditions to which they are exposed [11, 27, 32–35]. In accordance with this distinction, the terms intentional and incidental will be used with reference to the environmental conditions under which learning is taking place without making any assumptions about the underlying language processes.

While a series of studies on artificial and semi-artificial languages has demonstrated a significant advantage of learning novel grammatical structures under intentional over incidental exposure conditions [24, 35–37], learners have been shown to succeed not only in learning grammatical structures incidentally, but, in some cases, in developing knowledge that is partly implicit (e.g., [13, 38, 39]). Although these findings may suggest that some aspects of L2 grammar can be learned without intention or awareness, the overall learning effect observed is generally limited. For example, Rebuschat and Williams [30] exposed adult learners to an artificial language consisting of English vocabulary and German word order and evaluated learning of word order via a grammaticality judgement task (GJT). Participants in the incidental group only performed with ~55% accuracy, while the addition of an elicited imitation task resulted in an increased learning effect (~62%). Subsequent studies have found similar learning effects [23, 24]. Small learning effects have also been reported in studies targeting the learning of novel case markers. Rogers et al. [13] tested L1 English speakers’ ability to learn case-marking incidentally by presenting them with a semi-artificial language consisting of English phrases and Czech nouns marked either for nominative (-a) or accusative case (-u). This design was aimed at directing participants’ attention to the target grammatical markers, thereby facilitating noticing. Despite that, and although learners showed above-chance performance, mean accuracy was only ~56%. These results have been corroborated by subsequent studies testing inflectional morphology learning under incidental exposure [12, 40, 41].

One potential explanation for these findings relates to the limitations imposed on learners by the nature of implicit learning. Incidental contexts are thought to engage primarily implicit learning processes [42]. According to the literature on L2 acquisition, the ability to learn a language implicitly decreases with age [16, 43]. However, this age effect does not apply uniformly to all components of implicit learning [44]. Therefore, adults may retain the ability to learn simple structures implicitly but may face problems with low-salience and abstract linguistic patterns and rules [45], which may explain learners’ severe difficulties with L2 inflectional morphology.

Additionally, implicit learning requires extensive and repeated exposure to input for the development of new linguistic knowledge [1]. Despite this, most studies examine learning outcomes shortly after exposure to novel structures, which may be too brief for robust learning to occur [14]. Importantly, prior work has shown that a mere increase in exposure to the linguistic stimuli over the same experiment does not significantly improve learning gains [41, 46]. In contrast, accuracy scores tend to improve when the length of exposure is extended to at least a second session [18]. One explanation for these different findings may be found in sleep-related memory consolidation of new information. Such memory effects have been demonstrated both for cognitive abilities, such as implicit learning [47], and for novel word learning [48]. Hence, it is likely that the findings of studies examining grammar learning after minimal exposure to input may underestimate adults’ learning abilities. Thus, given the scarcity of (micro-)longitudinal studies in this area, the extent to which adults can learn grammatical rules under incidental conditions is still unclear, and so is whether receiving relatively extensive incidental exposure would result in higher levels of L2 grammatical accuracy. To gain a fuller understanding of adult learners’ capacity for incidental grammar learning, in the present study, language exposure was spread over five separate sessions, which allowed us to track how learning develops over time.

### L1 transfer in L2 grammar learning

Another reason for the meagre learning effects observed in previous studies may be tied to the fact that the acquisition of various aspects of grammar is generally particularly challenging for late L2 learners [49–51]. The cause of these difficulties can be traced to various factors, such as input frequency [52, 53], complexity of features [54], emotions [55], individual differences in cognitive abilities [56] and prior L1 knowledge [57–59]. Among these factors, understanding how prior linguistic experience can influence the perceived difficulty of L2 structures has received considerable attention in the L2 learning literature. Previous research has shown that, at the initial stages of learning a new language, participants tend to use L1 processing strategies to interpret L2 sentences [60–63]. Prior L1 experience tunes the perceptual system interfering with subsequent L2 processing. Associative learning mechanisms are, thus, hindered by such learned attention effects [58, 64]. Specifically, earlier L1 experience with a cue (e.g., a temporal adverb such as *yesterday* or *today*) that reliably leads to a particular outcome (e.g., temporal reference) may block the acquisition of another cue that is also relevant for the interpretation (e.g., past tense *-ed*) [65]. Such effects can be particularly detrimental for cues that lack perceptual salience, have low communicative value (e.g., agreement) and are not present in the L1.

However, studies directly addressing the effect of L1 experience on artificial language learning are scarce. Some evidence of this effect comes from Williams and Kuribara [25], who exposed L1 English speakers to a semi-artificial language consisting of English words and Japanese syntax. Participants in the incidental exposure condition were informed about the function of the case markers and were presented with a number of sentences, including mainly canonical sentences and a minority of scrambled structures. They were then tested on their ability to learn the different word order regularities of the language. The results of a GJT containing novel lexis and some new structures showed that while participants learned the canonical structures, they did not reliably reject the new ungrammatical sentences, particularly those that were grammatical in English, indicating that they did not generalize the notion of scrambling to new sentences. Instead, learners developed a strong preference for canonical word orders. Additional evidence is provided by Gao and Ma [35]. In a replication of the Tagarelli et al. [24] study, L1 Chinese participants were presented with sentences that had Chinese vocabulary and German grammar, allowing for three grammatical structures, one simple and two complex structures, which differed in terms of verb placement. Following exposure, participants trained in both incidental and instructed conditions completed a GJT and an elicited production task. While in the original study the incidental exposure group of L1 English speakers learned both the simple and one of the complex patterns, linguistic complexity did not emerge as a significant predictor for the L1 Chinese speakers, who performed close to chance on all structures. According to the authors, performance can be attributed to the fact that Chinese allows for verbs to occur later in the sentence, causing strong L1 interference. Similar findings appear to emerge from studies examining incidental learning of mappings between novel determiners and semantic properties of nouns. In one of their experiments, Leung and Williams [66, 67] introduced L1 Chinese and L1 English speakers to a miniature artificial determiner system and instructed that the determiners encode the distance between the speaker and the object (*gi* and *ro* for near objects and ul and *ne* for far objects). However, they were not informed that these determiners also referred to the shape of objects (*gi* and *ul* referred to long objects while *ul* and *ne* referred to flat objects). Subsequently, participants were tested on their ability to incidentally learn the relationships between the determiners and their shape meanings. Both groups were visually presented with noun phrases (e.g., *gi shoelace* vs *ul tissue*) in their native language and were asked to indicate, as quickly and accurately as possible, first whether the object presented was long or flat and, secondly whether the object was near or far. L1 Chinese speakers, but not L1 English speakers, managed to learn the hidden associations, taking advantage of the fact that the shape distinction is explicitly encoded in the classifier system of (written) Chinese. Using the same artificial determiner system and experimental design, Cayado and Chan [67] tested Chinese–English bilinguals’ and native English speakers’ ability to learn the associations between determiners and fire/water semantic categories (*gi* and *ul* for water-related words and *ro* and *ne* for fire-related words), a distinction that is also marked in written Chinese. Here, test items were presented in English to both groups. While both groups showed evidence of learning, Chinese–English bilinguals responded faster than the L1 English speakers despite testing taking place in their L2. Thus, overall, earlier studies suggest that different patterns of performance may arise depending on learners’ L1 background. Yet, to date, the role of L1 experience and transfer in artificial language has not been comprehensively tested, limiting the generalizability of previous findings as well as the potential and limits of adult incidental grammar learning. Therefore, an additional aim of the current study was to remedy this by investigating how prior linguistic experience moderates L2 grammar learning under incidental exposure conditions.

### Artificial language paradigms

Natural languages are highly complex; consequently, isolating and examining how learners acquire a particular structure or pattern and what factors are involved in the acquisition process can be a difficult endeavor. This problem can be overcome by using artificial language paradigms [for reviews, see 68, 69]. The use of such paradigms allows for exerting full control over the type of structures or patterns to be tested, the degree of (di)similarity to learners’ known language(s), the amount of input that learners are exposed to and the type of exposure. In contrast to natural languages, artificial languages allow participants to achieve high levels of proficiency within a short amount of time. Furthermore, given that the vast majority of artificial languages studies is conducted within a controlled laboratory environment, researchers are afforded the opportunity to specify the desired inclusion criteria and focus on specific structures, while also avoiding potential confounds associated with the characteristics of the participants [70].

However, the use of these paradigms does have some potential limitations, the major of which is likely the concerns over their ecological validity. This is because, given the simplified nature of the input and the target structures, the results from artificial languages may not fully scale up to natural languages. This seems to be particularly relevant for semi-artificial languages, where the insertion of artificial or unknown morphological markers to real words, often known by learners, may increase the salience of these markers, and consecutively their learnability [27, 33]. Despite these concerns, previous neuroimaging studies suggest the existence of significant parallels in the brain activity during artificial and natural language processing [71–73]. Additionally, performance on artificial language learning measures has been found to correlate positively with accuracy on natural language learning measures [74]. Therefore, the methodological advantages that these artificial language paradigms offer and the similarities in the neural correlates and mechanisms underlying processing of novel and native language constructions allow such paradigms to serve as ‘test tube’ models of natural languages [38], thus, making them a particularly useful tool to investigate L2 leaning and bilingualism [75, 76].

#### The present study

The primary goal of the current study is to investigate whether adults can learn different aspects of novel language grammar under incidental exposure conditions. Extending earlier work, we explore whether prolonged incidental exposure can lead to more robust learning effects. Additionally, we examine the extent to which grammar attainment is influenced by learned attention effects stemming from learners’ prior L1 experience. The findings of two experiments are reported here. In the first experiment, adult L1 English speakers were exposed to an artificial language over five separate sessions, during which they were trained on the vocabulary of the language and completed a series of grammatical comprehension tests. The artificial language, Kepidalo, had variable word order and case marking on nouns and adjectives, features that are not present in English. To further tease apart the effects of incidental exposure and prior L1 experience on grammar learning, in a follow-up study (i.e., Experiment 2) we repeated the same experiment, but this time with native speakers of German, a morphologically richer language, while also increasing the amount of exposure to six sessions. In addition, Experiment 2 also investigates the relationship between learning outcomes and participants’ level of metalinguistic awareness, which was assessed by means of a post-test questionnaire.

The research questions addressed in the following experiments were the following:

RQ1. Can adult learners acquire grammar under incidental exposure conditions?
RQ2. If so, what aspects do they acquire (word order, case marking, agreement marking)?
RQ3. Is extensive incidental exposure sufficient to obtain robust learning effects?
RQ4. To what extent does learners’ L1 background modulate L2 grammar learning?
RQ5. To what extent is L2 grammar learning associated with metalinguistic awareness of the target structures?

Regarding RQ1, based on previous research demonstrating at least some grammar learning after a single session of incidental exposure to the linguistic stimuli (e.g., [12, 13, 23, 40]), and considering the extensive amount of artificial language input participants received, we predicted that evidence of grammar learning would be found for both L1 groups. For RQ2, following previous studies, we hypothesized that both the L1 English and the L1 German participants would show greater learning effects for word order than for morphology, given the low salience of morphosyntactic cues. For RQ3, given the scarcity of available research, our predictions are more tentative. While both Rogers [40] and Williams [46] failed to find better performance after increasing or even doubling the amount of exposure to stimuli within the same session, Pili-Moss, Brill-Schuetz, Faretta-Stutenberg and Morgan-Short [77], who provided a session-by-session analysis of the data originally collected in Morgan-Short et al. [18], found that learners’ grammatical abilities improved over time (see also [78] for a similar pattern of results). Given that in the present study participants completed each session on different days, allowing for consolidation effects to occur, we hypothesized that grammatical comprehension would increase as a function of time. Despite that, we still expected persistent difficulties with inflectional morphology throughout the study. Regarding RQ4, it was predicted that the L1 English learners would show strong L1 transfer effects leading to relatively low accuracy scores at the early stages of exposure, with performance then improving as a function of time (experiment 1). Since the L1 German participants have prior experience with case marking and word order variation from their L1, we expected them to outperform the L1 English group on all aspects of grammar (experiment 2). Finally, for RQ5 (experiment 2), we hypothesized that the development of knowledge of which learners are aware would result in greater learning outcomes, given its facilitative role in L2 learning [17, 39, 79–81]. In addition, it was expected that the effect of metalinguistic awareness on learning would become stronger with increased exposure to artificial language input. Predictions regarding RQs 1, 2, and 5 were borne out, while our results regarding RQs 3 and 4 were less conclusive.

## Experiment 1

### Method

#### Participants

Forty-one adults with a mean age of 22.02 years (SD = 4.17, range = 18–35) participated in the study. All participants were monolingual native speakers of English who were resident in the United Kingdom. Recruitment was conducted via email and social media (Facebook and Twitter; N = 14) and through Prolific, an online participant recruitment platform (https://www.prolific.co; N = 27). To ensure that participants fulfilled the inclusion criteria, the following filters were applied: *English speaking Monolingual*, *Nationality*: *United Kingdom*, *Country of Birth*: *United Kingdom*, *Age*: *18–35*, *Country of Residence*: *United Kingdom*. Participants were asked to electronically consent to take part in the study and received monetary compensation for their time (60.70 GBP). Based on self-reports, participants had on average 15.93 years of formal education (SD = 1.79, range = 12–21).

#### Artificial language learning game

Participants were exposed to the novel artificial language in the context of an online computer-based game. In this game, the learners’ task was to travel to Tikon, a fictitious galaxy, and complete a number of challenges in order to collect four weapons that would help them defend the earth from an alien invasion. In order to accomplish their goal, participants had to learn an artificial language, namely Kepidalo.

The lexicon of Kepidalo comprised 14 disyllabic pseudowords: 8 nouns, 4 verbs and 2 adjectives (see S1 Appendix). The verbs designated semantically transitive events and always occurred with a direct object. All nouns and adjectives were overtly marked for case. The nouns were evenly distributed into two classes. The nominative case of nouns belonging in Class 1 was marked with the suffix *-i*, whereas nouns of Class 2 bore the suffix *-a*. In the accusative case, nouns of both classes took the suffix *-o*. The novel words were constructed to be easily pronounceable by participants.

In terms of syntax, Kepidalo was a verb-final language in which the order of subject and object was free, thus exhibiting either a canonical (SOV; 1a) or a non-canonical (OSV; 1b) word order. Adjectives were optional, occurred postnominally and carried an inflection morpheme that agreed in class and case with the noun they modified.

(1) a. Noun_NOM_−(Adj_NOM_)–Noun_ACC_−(Adj_ACC_)–Verb
  b. NounACC−(Adj_ACC_)–Noun_NOM_−(Adj_NOM_)–Verb

A total of 400 Kepidalo sentences were generated for the purpose of the experiment. Within these sentences, all lexical items (nouns, verbs and adjectives) occurred an equal number of times, with each noun being assigned to the subject and the object positions with equal frequency (45 times in each position). In addition, 290 of the sentences were SOV while the remaining 110 sentences had a non-canonical OSV word order. All sentences were three to five words long and had an average duration of 1967 ms (range = 1622ms– 2742ms). The auditory stimuli were synthesized using the Google Cloud Text-to-Speech service. We opted for the use of a Polish accented synthesized voice to contribute to the impression that participants were learning a new language spoken by an alien character. Furthermore, a slightly slower than the normal speaking rate was employed (0.75 with 1 being the normal), as this is thought to facilitate L2 comprehension [82, 83].

The novel sentences described the actions of eight alien cartoon characters that corresponded to the eight nouns in the artificial language. The aliens appeared in two different colors, dark red or light green, each of which corresponded to one of the two Kepidalo adjectives. Short animated scenes in which the aliens were seen performing one of four simple actions (approaching, catapulting, chasing, or jumping over) were generated and were then converted to GIF format. The reason for using GIFs instead of videos was twofold: first, they are small in size, thus taking less time to load even on devices with slower network connections [84]. Second, GIFs’ ability to loop continuously allows participants to spend as much time as they need to while processing the stimuli while also minimizing the need to interact with the device during playback [85].

#### General procedure

The experiment was conducted online through the Gorilla experiment builder [gorilla.sc; 86] and could only be accessed via computers and laptops. Participants were, first, asked to electronically fill out a short background questionnaire concerning their demographics and prior language experience. Those who met all the inclusion criteria individually participated in five separate sessions within a 10-day span. The time interval between sessions was at least 24 hours but not more than 48 hours. The experimental stimuli and scripts for all tasks used in this study are available on Gorilla Open Materials (https://app.gorilla.sc/openmaterials/484592).

A summary of the tasks that participants had to complete in each session, and the order in which they were administered is provided in Table 1. Over the first four sessions, participants were trained on the vocabulary of the novel language and were tested on their knowledge of the language’s grammar (word order, case marking). During the final session, two additional tasks designed to probe grammatical knowledge were administered. Each session also included a cognitive test measuring individual differences (not discussed here). The order of presentation of the tasks was the same for all participants.

**Table 1:** Summary of the artificial language tests administered during the study.

Session 1	Session 2	Session 3	Session 4	Session 5
1. Pretraining				1. Final Grammatical Comprehension test
2. Lexical Training	1. Lexical Training	1. Lexical Training	1. Lexical Training	2. Grammaticality Judgment task
3. Grammatical Comprehension test	2. Grammatical Comprehension test	2. Grammatical Comprehension test	2. Grammatical Comprehension test	

#### Pretraining

The first session of the study began with training on the nouns of the novel language. Participants were told that, as part of their mission, they would need to learn the names of the inhabitants of the Tikon galaxy. A four-alternative forced-choice (4AFC) task modeled after Llompart and Reinisch [87, 88] was used to assess learning of the lexical items. The task contained 2 identical phases: a training phase consisting of 64 familiarization trials, and a test phase during which each of the 8 nouns was presented twice, for a total of 16 trials. In each trial, four aliens of the same color were presented simultaneously, one in each corner of the screen. When participants clicked on a “Play” button, the name of one of the aliens was presented auditorilily in the nominative form (e.g., *Alg-i*, *Flub-a*). The participants’ task was to choose the picture that matched the word they had just heard. Visual feedback on accuracy was provided in the form of a green tick for correct answers or a red cross for incorrect answers. Following this, the target alien appeared on the screen in isolation for 1500ms and the corresponding noun was presented auditorily again. The location of the target items on the screen was randomized and the same trial sequence was used for all participants.

The familiarization phase was mandatory for all participants and the test phase differed in that participants’ scores determined whether they were ready to proceed to the next phase. Participants who achieved 100% accuracy on the test trials proceeded immediately to the next task, while those who failed to achieve the target score (n = 16) were given an additional 16 trials practice. After these additional trials, all participants were allowed to move on to the next task regardless of their final score.

#### Lexical training

During each of the first four sessions, participants were exposed to 270 auditorily presented Kepidalo sentences, which were pseudo-randomly selected from the total set of 400 sentences that were originally generated. To control for possible recency and primacy effects, four randomized lists were created, one for each session. The sentences were divided into three training blocks, each containing 90 sentences. The order in which the sentences appeared was the same across participants. To keep participants motivated throughout the task, they were told that each correct answer would award a unit of solar energy which would propel them towards the next planet in the game, whereas each incorrect would decrease their solar energy by 1 unit. All participants advanced to the next task, regardless of the number of correct answers.

**Fig 1:**
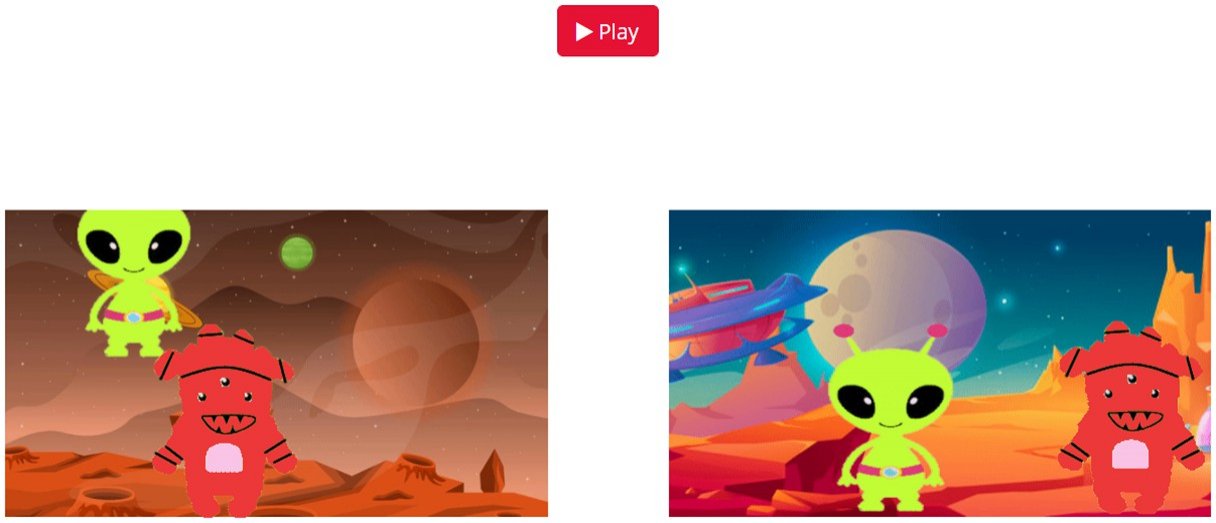
Screenshot of a training trial in the lexical training task (Left scene: *The (green) velg is jumping over the (red) prad*, Right scene: *the (green) velg is approaching the (red) prad)*.

Lexical training involved a two-alternative forced-choice task (2AFC). In each trial two short animated scenes, each showing two aliens performing an action, were simultaneously displayed on the screen (Fig 1), while a sentence describing the events depicted in one of the two scenes was played **(2)**. Participants were instructed to indicate, as quickly and accurately as possible, which of the two scenes the sentence described. Participants could replay the sentence a second time if they wished, and the animated scenes looped continuously until they responded. The side of the target scene was counterbalanced and participants received visual and auditory feedback immediately after they provided a response. At the end of each of the three blocks, a display showing participants’ cumulative score was presented.

(2) Velg-a pog-a prad-o kov-o varek
  velg-NOM green-NOM prad-ACC red-ACC jump-over
  the green velg jumps over the red prad

Crucially, the target and distractor scenes were designed to differ by one single element. Specifically, in trials testing knowledge of verbs, the target and distractor scenes differed in terms of the action that the aliens performed; in the noun test trials, the two scenes differed with regards to one of the alien characters; finally, in the trials testing adjective learning, the color of one of the aliens in the distractor scene was changed. There were 90 trials for each of the three lexical categories involved (verbs, nouns, adjectives) interspersed among the three training blocks. The majority of the sentences had a canonical SOV word order (200), while the rest were OSV (70).

#### Grammatical comprehension test

At the end of the lexical training trials, participants were tested on the grammar of the artificial language by being exposed to 90 new sentences. Seventy of those sentences displayed SOV word order and 20 were OSV. Participants heard the same 90 sentences over the four sessions. However, a different pseudorandom order was created for each session. All participants saw the stimuli in the same sequence.

A two-alternative forced-choice task (2AFC) was used for testing grammar learning and the procedure followed was similar to the one used for lexical training; participants heard a sentence in the artificial language and viewed two animated scenes: a target scene which depicted the meaning of the sentence and a distractor scene in which the agent and patient roles of the two aliens were reversed. No feedback regarding accuracy was displayed. The side of the target video (left or right) was counterbalanced within each list.

#### Grammaticality judgement task

In the final session, grammatical knowledge was assessed by means of a Grammaticality Judgement Task (GJT) in which participants were presented with novel sentences (i.e., sentences that were not used in the preceding sessions) and were asked to decide whether the sentences were correct or incorrect. The GJT task consisted of 80 sentences. Half of the sentences were grammatical and the other half contained various kinds of grammatical violations (Table 2). Violations of each type occurred 8 times each.

**Table 2:** Types of ungrammatical sentences in the grammaticality judgement task.

Violation type	Example	Explanation
Verb placement	1. Fluba kova **varek** olbo pogo. 2. ***Dolek*** irda flubo.	Verb in sentence-medial (1) or sentence-initial position (2)
Nominative marking	Prad**i** flub**a** varek.	Both nouns carry nominative marking
Accusative marking	Velg**o** urg**o** dolek.	Both nouns carry accusative marking
Adjective noun agreement	1. Pradi olb**o** kov**a** birek. 2. Alg**i** kov**a** Torgo varek.	Adjective modifying the object carries nominative marking
Adjective placement	***Kova*** velga olbo mulek.	Adjective in prenominal position

SOV and OSV patterns appeared with equal frequency during the task. Within each construction, i) half of the sentences were grammatical and half ungrammatical, and ii) half of the sentences included an adjective which modified the subject (10) or the object (10) of the sentence. The sentences appeared in random order, but the order of presentation was the same for all subjects. No feedback was given on responses.

#### Final grammatical comprehension test

In the final task, participants were once again tested on their knowledge of the grammar of the artificial language by means of a 2AFC task that was identical to the Grammatical Comprehension Test blocks administered in the previous sessions. The auditory stimuli consisted of the same 40 grammatical sentences that were presented in the GJT, thus SOV and OSV appeared equally frequently. Each sentence was accompanied by a target video that correctly depicted the sentence and a distractor video showing reversed subject/object roles.

### Results

#### Data analysis

For the Lexical Training and the Grammatical Comprehension test, mean accuracy scores and mean reaction times were calculated for each individual for each of the four sessions and were then averaged across participants. Performance is summarized in Table 3. For the Pretraining task, individual scores were calculated as the total number of correct responses during the training phase and the first test block (80 items in total). For the GJT task, following signal detection theory [89], participants’ ability to discriminate between correct and incorrect sentences was measured by d-prime (d’). Specifically, four scores were obtained for each participant: hits (grammatical sentences judged as acceptable), misses (grammatical sentences judged as unacceptable), false alarms (ungrammatical sentences judged as acceptable) and correct rejections (ungrammatical sentences judged as unacceptable). From these scores, d’ scores were calculated for each participant [90] using the ’psycho’ package [version 0.6.1; 91] in RStudio [92]. A d-prime score of 0 indicates chance performance and high d’ scores indicate greater discrimination sensitivity. Finally, for the Final Grammatical Comprehension test (henceforth, FGCT), individual scores were calculated as the number of correct responses provided by each participant. A summary of participants’ performance on the three artificial language tasks is presented in Table 4. Correlation matrices showing the relationship between the three artificial language tasks and performance on the Lexical Training and Grammatical Comprehension trials in each session are provided in the S2 Appendix. Data from each task (except Pretraining) were analyzed separately using mixed-effects models. To further explore significant interactions, post-hoc pairwise comparisons were performed using the emmeans package [version 1.8.1.1; 93]. Finally, we calculated effect sizes for the models, measured by marginal and conditional R^2^, using the rsquared.GLMM function from the MuMIN package [version 1.47.1; 94], odds ratios and confidence intervals for the predictor variables using the tab_model function from sjPlot package [version 2.8.11; 95] and Spearman-Brown split-half reliability for all test measures using the splithalf package [version 0.8.2; 96]. These reliability coefficients are reported in S3 Appendix. All data and R scripts for the analyses are available at (https://osf.io/3jy52/?view_only=2664569e74964a5b84c2c3989f70e41f).

**Table 3:** Mean accuracy and reaction times across sessions in the lexical training and grammatical comprehension blocks.

	Lexical Training^1^			Grammatical Comprehension test^2^		
	M%	Accuracy M (SD)	Reaction Time M (SD)	M%	Accuracy M (SD)	Reaction Time M (SD)
Session 1	68.9	186.1 (32.7)	5217.0 (1782.2)	62.7	56.4 (11.1)	3686.0 (1428.6)
Session 2	79.4	214.3 (34.7)	4158.2 (1316.1)	64.4	58.0 (10.8)	2952.3 (854.4)
Session 3	82.4	222.6 (37.7)	3645.1 (1049.6)	64.4	58.0 (9.8)	2498.9 (794.0)
Session 4	84.9	229.1 (35.1)	3468.6 (1206.5)	67.0	60.3 (12.1)	2164.5 (800.2)
Overall	78.9	213.0 (38.5)	4122.2 (1516.1)	64.6	58.2 (11.0)	2825.4 (1148.4)

^1^ Accuracy scores were calculated out of a maximum of 270.

^2^ Accuracy scores were calculated out of a maximum of 90.

**Table 4:** Descriptive statistics for performance on the pretraining and post-tests grammar tasks.

	M (SD)	Median	IQR	SE	Range
Pretraining^1^	61.83 (9.36)	62.0	12.0	1.46	32–74
Grammaticality Judgement Task (*d’*)	1.23 (0.64)	1.28	0.95	0.1	0.12–3.09
Final Grammatical Comprehension test^2^	21.37 (4.18)	20.0	1.0	0.65	17–40

^1^ Accuracy scores were calculated out of a maximum of 82.

^2^ Accuracy scores were calculated out of a maximum of 40.

#### Lexical training

Since Language Training scores were obtained from a 2AFC picture selection task, chance-level performance was 50% or 135 correct responses (out of 270 trials). Participants’ performance was greater than chance from the first session onwards and their ability to discriminate between correct and distractor scenes continued to improve throughout the study.

With regards to performance by distractor type, accuracy was higher on trials involving noun distractors (*M* = 88%, *SD* = 8.1%), followed by trials including verb (*M* = 79.8%, *SD* = 15.7%) and adjective distractors (*M* = 68.9%, *SD* = 18.2%). Regarding Word Order, participants achieved similar accuracy for SOV (*M* = 79.2%, *SD* = 11.9%) and for OSV sentences (*M* = 77.9%, *SD* = 12.4%).

To determine how accuracy rates on the lexical training task changed as a function of time and whether the presence of pretrained words affected learning, trial-by-trial data from the Lexical Training trials were submitted to a mixed-effects logistic regression model using the lme4 package [version 1.1–30: 97] in R. This type of model is well suited to analyzing binary response data [98, 99]. Response accuracy, coded as correct (1) or incorrect (0), was entered as the categorical dependent variable. Pretraining score was included in the model as a continuous variable. Scores from the Pretraining task were centered and scaled using the scale() function in R. Session (contrast coded as -1, -0.5, 0.5 and 1, for Sessions 1 to 4, respectively) and Word Order (contrast coded as -0.5 and 0.5, for OSV and SOV respectively) were also entered in the model as predictors. Contrast coding allows for recentering categorical variables by making the intercept the grand mean (i.e., 0), so that the predictors and their interactions can be interpreted in a manner analogous to ANOVA. By doing so, the direction of the overall effect of predictors in the model is indicated by the regression weights (positive or negative). The model contained all the two-way interactions between the predictors. The predicted probabilities of correct responses for all contrasts of interest were computed using the ggeffects package [100; version 1.1.3].

Data were initially fitted to a model containing random intercepts for participants and items. To determine the best random-effects structure, random slopes for all fixed effects were first tested separately and then compared to the random intercepts only model by means of likelihood ratio tests using the *anova()* function of R. Subsequently, random slopes were added to the model one at a time, starting from the one that improved the model’s fit the most and were retained if the model converged and fitted the data significantly better than the previous base model (the model without the random slope) as determined by likelihood ratio tests. The best-fitting model contained random intercepts for participants and items, by-participant random slopes for Session and by-item random slopes for Pretraining. The output of the best-fitting model is shown in Table 5.

**Table 5:** Mixed-effects model fitted to the lexical training data.

Variable	*B*	*SE*	*Z*	*P*	*Odds Ratios (CI)*
Intercept	1.862	0.386	4.819	< .001	6.44 (3.02–13.72)
Session	0.776	0.116	6.680	< .001	2.17 (1.73–2.73)
Word Order	0.091	0.067	1.353	.176	1.10 (0.96–1.25)
Pretraining	0.580	0.166	3.495	< .001	1.79 (1.29–2.47)
Session: Pretraining	0.189	0.115	1.652	.099	1.21 (0.97–1.51)
Session: Word Order	0.004	0.037	0.110	.912	1.00 (0.93–1.08)
Word Order: Pretraining	0.019	0.030	0.627	.531	1.02 (0.96–1.08)
*Random effects*	Variance	SD			
Item: Distractor Type (intercept)	0.183	0.428			
Participant (intercept)	0.977	0.988			
Distractor Type (intercept)	0.377	0.614			
Item: Distractor Type | Pretraining (slope)	0.010	0.101			
Participant | Session (slope)	0.525	0.724			
Distractor Type | Pretraining (slope)	0.010	0.10			
Marginal R^2^	.126				
Conditional R^2^	.444				

**Fig 2:**
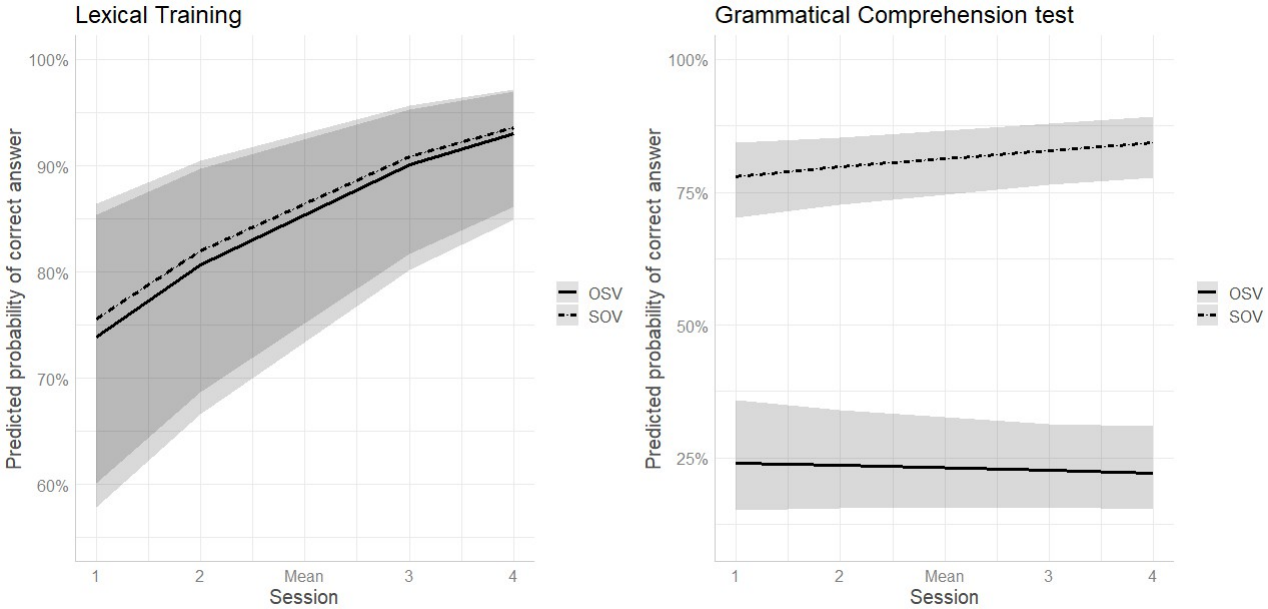
Predicted probability of a correct answer as a function of Session and Word Order in Lexical Training (left) and Grammatical Comprehension test (right) in L1 English participants.

The model revealed significant effects of Session and Pretraining, which as indicated by the positive coefficients suggest that vocabulary learning rates improved significantly across sessions and that learners who exhibited better learning of the lexical items in the Pretraining task were more likely to achieve higher accuracy in the Lexical Training trials. Furthermore, the interaction between Session and Pretraining, albeit not significant, suggests that the effect of Session was stronger for participants with higher Pretraining scores. Finally, performance did not differ significantly between the two Word Orders and there was no interaction between Session and Word Order as leaners appeared to respond with approximately equal accuracy to both types of sentences across sessions (Fig 2). Specifically, the estimated accuracy was 87% for SOV and 86% for OSV sentences.

#### Grammatical comprehension test

For each Session of the task, 50% or 45 correct responses (out of 90 trials) represent chance-level performance. As shown in Table 3, accuracy in the grammatical comprehension trials was above chance across all sessions, but performance remained stable over time. With regards to Word Order, overall, performance was better on SOV (M = 74.7%, SD = 15.8%) than on OSV sentences (M = 29.4%, SD = 16.7%).

A mixed-effects logistic regression model was fitted with Accuracy (correct = 1, incorrect = 0) as categorical dependent variable and with Session and Word Order, contrast coded in the same way as described in the model on Lexical Training data above, as predictors. In order to examine whether participants’ initial knowledge of words affects grammar learning, Pretraining was also entered as predictor. Furthermore, the model included all the two-way interactions between the three variables. The random-effects structure was selected following the process outlined above. The final model contained random intercepts for participants and items, by-participant and by-item random slopes for Session and by-participant slopes for Word Order.

According to the model (Table 6), the effect of Session was not significant, suggesting that, despite an improvement in performance as shown by the positive estimate of the effect, overall, learners’ accuracy rates did not increase substantially over time. However, there was a significant effect of Word Order and a significant interaction between Word Order and Session, qualifying the main effect of Session. The positive coefficient for the Word Order effect suggests that participants were more accurate on SOV than on OSV sentences, corresponding to an estimated accuracy of 81% and 23% respectively, and a follow-up analysis on the interaction revealed that the effect of Session was significantly higher for SOV sentences as compared to OSV ones, indicating that the difference in performance between the two Word Orders increased across sessions (Fig 2).

**Table 6:** Mixed-effects model fitted to the grammatical comprehension test data.

Variable	*B*	*SE*	*Z*	*P*	*Odds Ratios (CI)*
(Intercept)	0.137	0.100	1.363	.173	1.15 (0.94–1.40)
Word Order	2.678	0.392	6.828	< .001	14.56 (6.75–31.40)
Session	0.077	0.087	0.886	.376	1.08 (0.91–1.28)
Pretraining	-0.003	0.039	-0.067	.947	1.00 (0.92–1.08)
Word Order: Session	0.263	0.087	3.021	.003	1.30 (1.10–1.54)
Word Order: Pretraining	0.472	0.346	1.364	.173	1.60 (0.81–3.16)
Session: Pretraining	0.012	0.079	0.146	.884	1.01 (0.87–1.18)
*Random effects*	Variance	SD			
Item (intercept)	0.513	0.717			
Participant (intercept)	0.039	0.198			
Item | Session (slope)	0.052	0.228			
Participant | Session (slope)	0.225	0.475			
Participant | Word Order (slope)	4.802	2.191			
Marginal R^2^	.201				
Conditional R^2^	.498				

#### Grammaticality judgement task

Performance on the task is summarized in Table 7. Overall, learners judged 64% (*SD* = 7.2%) of the test sentences correctly, but performance was driven primarily by accuracy on grammatical rather than on ungrammatical test sentences. The descriptive statistics show variation in performance on different types of violation, with participants achieving higher scores on sentences involving word order violations than on sentences that contained case marking violations. Performance on sentences with SOV and OSV word order was nearly indistinguishable (*M* = 64.5%, *SD* = 6.5%, and *M* = 63.6%, *SD* = 9.3%, respectively).

**Table 7:** Mean percentage of correct responses (SDs) and d’ scores by sentence type the grammaticality judgement task.

Condition	Grammatical	Ungrammatical	d’
	M (SD)	M (SD)	M (SD)
*Word order violations*			
Verb placement	89.9 (14.6)	99.4 (2.7)	2.75 (0.56)
Adjective placement	87.8 (21.6)	47.6 (18.0)	1.11 (0.72)
*Case marking violations*			
Nominative marking	91.8 (11.4)	12.8 (20.6)	0.13 (0.57)
Accusative marking	92.7 (15.6)	11.0 (19.6)	0.15 (0.66)
Adjective noun agreement	90.9 (13.4)	16.5 (26.0)	0.21 (0.78)
Overall	90.6 (12.5)	37.4 (14.4)	1.63 (0.93)

In order to assess the extent to which participants learned the syntactic structure of the artificial language, data were submitted to a mixed-effects logistic regression model with Accuracy as a binary outcome variable (correct = 1 vs incorrect = 0) and Word Order (contrast coded with OSV as -0.5 and SOV as 0.5), Pretraining, Grammaticality (contrast coded with ungrammatical sentences as -0.5 and grammatical as 0.5) and Error Type (contrast coded with case marking sentences as -0.5 and word order sentences as 0.5) as independent variables. All the two-way interactions between the aforementioned predictors were also entered in the full model. The model included random intercepts for participants and items, by-participant random slopes for Grammaticality and Error Type.

The model (See S4 Appendix for the full model) returned a significant effect of Grammaticality (*β* = 4.536, *z* = 6.844, *p* < .001), indicating that learners judged grammatical sentences more accurately than ungrammatical ones, as shown by the positive coefficient, with predicted accuracies of 98% and 38% respectively. There was a significant effect of Pretraining (*β* = 0.288, *z* = 2.003, *p* = .045), and a significant interaction between Grammaticality and Pretraining (*β* = 1.298, *z* = 2.675, *p* = .007) suggesting that participants with higher scores in the Pretraining task performed more accurately in this task and that the effect of Pretraining was different at different levels of the Grammaticality variable. Follow-up simple slope analysis, which involves estimating and comparing the slopes of the covariate trend for each level of a factor variable, showed that the effect of Pretraining was positive for grammatical sentences but negative and non-significant for ungrammatical items. Finally, the model showed a positive coefficient for the effect of Error Type (*β* = 2.940, *z* = 6.292, *p* < .001), suggesting that, overall, participants performed better on word order than case marking sentences, with 96% and 58% predicted accuracies respectively, and significant interactions between Grammaticality and Error Type (*β* = -4.466, *z* = -5.465, *p* < .001) and between Pretraining and Error Type (*β* = 0.438, *z* = 2.075, *p* = .038). Regarding the former interaction, post-hoc analyses revealed that the effect of Grammaticality, while significant for both types of sentences, was bigger for those in the case marking condition, with the predicted probability of a correct response increasing by 94% from ungrammatical (4%) to grammatical (98%), as compared to an increase of 10% for word order sentences (89% for ungrammatical and 99% for grammatical). For the latter of the two interactions, simple slope analysis found that the effect of pretraining was stronger and significant only for word order sentences.

#### Final grammatical comprehension test

Overall, participants exhibited high accuracy on SOV (M = 74.9%, SD = 18.6%), but were below chance on OSV sentences (M = 32%, SD = 23%). Yet another mixed-effects logistic regression model (See S4 Appendix for the full model) was built with Accuracy (correct = 1 vs. incorrect = 0) as a binary outcome, Pretraining, Word Order (contrast coded with OSV as -0.5 and SOV as 0.5) and their interaction as fixed effects, random intercepts for participants and items and random by-participant slopes for Word Order. A significant effect of Word Order was found (*β* = 2.522, *z* = 5.538, *p* < .001), which confirmed that participants reliably identified the correct picture for SOV items (81% predicted accuracy) but encountered difficulties doing so when presented with OSV sentences (26% predicted accuracy) even after four sessions of incidental exposure to the grammatical structure of the artificial language. Finally, the effect of Pretraining was not significant (*β* = 0.035, *z* = 0.374, *p* = .708), nor was the interaction between Pretraining and Word Order (*β* = 0.055, *z* = 0.137, *p* = .891).

### Discussion

As we have seen, our participants’ accuracy scores on the grammatical tasks were relatively low (*M* = 64.6% in the Grammatical Comprehension test; *M* = 64% in the GJT; *M* = 53.4% in the FGT), confirming that learning new grammatical constructions under incidental exposure conditions, without instruction about the structure of the language or feedback on accuracy of performance, is particularly challenging for adult L2 learners [cf. 13, 36, 80]. This difficulty seems to hold even after extensive exposure to the artificial language and despite the fact that participants performed relatively well on the lexical trials even in the early stages of the experiment. Specifically, while participants succeed in interpreting the SOV sentences, their ability to identify the correct scene upon hearing stimuli that had the less frequent OSV word order did not exceed chance level at any point during the study. This could be taken to suggest that participants might have relied primarily on word order cues, as opposed to inflectional morphology, in order to process the Kepidalo sentences. Further support for this interpretation comes from participants’ performance on the GJT. As shown in Table 7, learners were more accurate when presented with sentences that had word order violations, especially with those containing verb placement errors, than when asked to detect the grammaticality of sentences that contained case marking violations. These results are in line with similar recent artificial language learning studies [11, 12] showing that under incidental exposure conditions, adult learners are more likely to develop knowledge of word order than case marking rules. This difficulty with case marking could be at least partially attributed to the effects of learned attention [58]. Specifically, a common theme across these studies is that the participants targeted were native English speakers. Hence, learners’ prior L1 experience with English, a fixed word order language without case marking, may have driven them to look for word order cues when processing the novel sentences, which would in turn block the learning of the low-salient inflectional markers. Furthermore, the fact that they are required to learn word order patterns (i.e., SOV or OSV) that are different from the canonical word order of their native language could have also led them to focus their attention on this aspect of the language.

However, there is one piece of evidence indicating that participants might have learned more than their performance implies. Specifically, performance on the Grammatical Comprehension test shows that, although participants demonstrated superior performance on SOV compared to OVS items, they did not consistently apply the dominant word order to all sentences at any point during the task. Instead, as shown in Fig 2, the mean accuracy rates for the two word order patterns approximated the relative proportion of each pattern in the input participants received during the lexical training parts in each session (i.e., SOV = 74%, OSV = 26%). Interestingly, learners’ performance in the FGCT followed a somewhat similar pattern (SOV: *M* = 74.5%, OSV: *M* = 32%). These results could be taken to imply that learners were indeed sensitive to the presence of two distinct word order patterns, as well as their frequency of occurrence. This finding seems to be in accord with previous studies showing that adult L2 learners may be capable of learning the probabilities of occurrence of different patterns in the input without necessarily learning the grammatical forms [101].

## Experiment 2

As mentioned in the introduction, a large body of literature has shown that early adult L2 learning is heavily influenced by effects arising from prior L1 knowledge [58, 102, 103]. Such effects can lead learners to mistakenly engage L1-tuned processing affecting the route of L2 development. The results of Experiment 1 appear to be in line with these observations and warrant the question as to whether similar patterns of performance would be found for speakers of a language that relies primarily on case marking cues. In addition, note that, in Experiment 1, there was a small but non-significant increase in learners’ overall grammatical comprehension scores across sessions. Though this cannot be taken as a clear indication of improvement in learning, it seems to be consistent with the idea that incidental acquisition requires extensive amount to linguistic input [104]. Therefore, the slow learning rate seems to be partly attributable to difficulties associated with learning under incidental conditions per se.

In order to investigate the extent to which the results obtained in Experiment 1 stem from learners’ L1 experience and/or from the limited capacity to learn novel grammatical structures through incidental exposure, in Experiment 2, we examined whether the pattern of performance obtained for the L1 English participants could be replicated with native speakers of a language that is morphologically richer than English, namely German. In addition to this, Experiment 2 had two further aims. The first of these was to test whether additional incidental exposure would result in further incremental increases in performance or whether performance would stabilize at a suboptimal level. To do so, the amount of exposure was extended to six sessions, thus providing more opportunities for learning the rules of the language. Second, we also aimed at examining whether learning in incidental conditions depends on the learners’ ability to make explicit inferences about the grammatical structure of the language. While previous studies have focused on tracking the development of awareness during the test phase using source attributions and confidence ratings (e.g., [11, 32, 105]), here, we tested the extent to which learners’ metalinguistic awareness at the level of understanding [106], as measured by a post-test questionnaire, was predictive of vocabulary and grammar learning and we assessed whether this effect varied across sessions.

### Method

#### Participants

Thirty-eight participants, all native speakers of German and residents of Germany with a mean age of 25.45 (*SD* = 4.39, range = 18–35) took part in this study. Participants were recruited through Prolific (www.prolific.co), gave consent to participate electronically, and were paid €88 for their time. No participants reported having prior exposure to a language with a rich morphological system, such as Latin or Russian. On average, participants reported having 15.93 (SD = 3.10; range = 6–22) years of formal education.

#### General procedure

Participants were invited to take part in an online study consisting of six sessions, during which they were exposed to the same artificial language stimuli as in Study 1. The procedure was identical up to the point described in Study 1 (see Table 1). In Session 5, after completing the GJT and the FGCT, participants were once more asked to complete a Lexical Training task and a Grammatical Comprehension test. The same fours tasks were given again in Session 6, but in a different order, with the Lexical Training and Grammatical Comprehension test preceding the GJT and the FGCT. After completing these measures, a post-test questionnaire was administered, aimed at investigating participants’ explicit knowledge of the grammatical rules of the artificial language.

#### Metalinguistic awareness questionnaire

The questionnaire consisted of two parts. The first part comprised 6 questions. Participants were initially presented with a grammatical OSV sentence accompanied by a target and a distractor scene and were asked to select the scene described by the sentence and provide an explanation for their choice. Following that, participants heard five ungrammatical sentences, one for each type of violation sentence that occurred in the GJT (see Table 2) and were asked whether they could identify and explain the error in each sentence.

The second part of the questionnaire diverged from the traditional metalinguistic awareness tests, which typically consist exclusively of error corrections and explanations, as it included 45 True/False questions that were designed to probe participants’ explicit knowledge of the word order and case-marking rules of the artificial language (see Gorilla Open Materials).

### Results

#### Data analysis

Data obtained from the five artificial language learning tasks (Pretraining, Lexical Training, Grammatical Comprehension test, GJT, FGCT) were coded following the procedure outlined in Study 1. Tables 8 and 9 provide descriptive statistics for the L1 German group’s performance on the Lexical Training task and the Grammatical Comprehension test, and on the remaining measures, respectively. Overall accuracy scores up to session 4 are also provided to allow for a more direct comparison with scores obtained in Experiment 1. For the metalinguistic awareness questionnaire, data from each part were coded separately. Responses in the first part of the questionnaire were coded as correct if the learners identified the error involved in the sentence (e.g., Stimulus: ‘kovo Prado Algi mulek’ ADJ PLACEMENT VIOLATION, Response: *‘the adjective comes after the noun’*), while partial credit (0.5) was given if they supplied a partial explanation of the error (e.g., Response: *‘kovo is wrong at the beginning’*). All transcribed responses were coded independently by two of the authors (inter-rater reliability measured with Cohen’s kappa was 0.88) and any discrepancies were discussed and resolved through consensus. Participants could get a maximum of six points for this component of the questionnaire. For the second part, each response was coded as 1 if correct and 0 if incorrect. Following that, scores from the two parts were combined to obtain a total metalinguistic awareness score for each participant. Correlation matrices for all measures are reported (S2 Appendix) and Spearman-Brown split-half reliability coefficients for all test measures (S3 Appendix) are provided in the Supplementary Materials. As in Study 1, we fit a series of mixed effects models on the item-by-item responses on each task separately and significant interactions were followed by post-hoc pairwise comparisons using the emmeans package in R. Last, effect sizes (marginal and conditional R2), were computed with the rsquared.GLMM function from the MuMIN package, odds ratios and confidence intervals for the predictor variables with the tab_model function from sjPlot package, predicted probabilities with the ggeffects package and Spearman-Brown split-half reliability for test measures with the splithalf package. These reliability coefficients are provided in S3 Appendix.

**Table 8:** Mean accuracy (%) and reaction times across sessions in the lexical training and grammatical comprehension blocks for the L1 German learners.

	Lexical Training^1^			Grammatical Comprehension^2^		
	M%	Accuracy M (SD)	Reaction Time M (SD)	M%	Accuracy M (SD)	Reaction Time M (SD)
Session 1	73.0	197.2 (38.6)	5226.8 (1645.6)	61.6	55.5 (14.4)	3843.8 (1516.9)
Session 2	84.5	228.2 (39.1)	4472.0 (1267.4)	69.9	62.9 (13.5)	3287.2 (1219.3)
Session 3	87.4	236.0 (38.2)	4394.7 (1543.5)	75.7	68.1 (13.8)	3320.1 (1416.4)
Session 4	89.2	240.8 (37.5)	3402.0 (838.0)	74.3	66.9 (15.9)	2784.1 (1032.9)
Overall *(Up to Session 4)*	83.5	225.6 (41.6)	4373.9 (1495.4)	70.4	63.4 (15.1)	3308.8 (1350.0)
Session 5	89.5	241.6 (36.9)	3265.6 (813.4)	76.2	68.6 (14.4)	2597.1 (1190.9)
Session 6	89.3	241.1 (35.7)	3085.2 (690.7)	76.8	69.2 (14.7)	2519.6 (1030.2)
Overall	85.5	230.8 (40.5)	3974.4 (1413.0)	72.4	65.2 (15.1)	3058.6 (1320.1)

^1^ Accuracy scores were calculated out of a maximum of 270.

^2^ Accuracy scores were calculated out of a maximum of 90.

**Table 9:** Descriptive statistics for performance on the pretraining, post-tests grammar tasks, and the metalinguistic awareness questionnaire for the L1 German learners.

	M (SD)	Median	IQR	SE	Range
Pretraining^1^	62.6 (7.3)	63.0	8.75	1.19	46–75
Grammaticality Judgement Task 1 (*d’*)	1.63 (0.9)	1.39	0.78	0.15	0.44–4.5
Grammaticality Judgement Task 2 (*d’*)	1.79 (1.0)	1.74	1.05	0.16	0.12–3.8
Final Grammatical Comprehension test 1^2^	25.4 (8.5)	21.0	14.3	1.38	17–40
Final Grammatical Comprehension test 2^2^	26.2 (9.2)	21.0	18.8	1.50	18–40
Metalinguistic Awareness questionnaire^3^	35.0 (8.5)	33.8	12.9	1.37	20.5–50

^1^ Accuracy scores were calculated out of a maximum of 82

^2^ Accuracy scores were calculated out of a maximum of 40

^3^ Accuracy scores were calculated out of a maximum of 51

#### Lexical training

Average accuracy on the Lexical Training items was greater than chance from as early as the first session and showed an upward trend until Session 4, where it stabilized until the end of the study (Table 8). Accuracy rates were higher for noun (*M* = 90.2%, *SD* = 10.2%) and verb trials (*M* = 88.2%, *SD* = 14.3%), compared to trials testing adjective learning (*M* = 78.1%, *SD* = 18.3%). Furthermore, participants responded with equal accuracy regardless of the Word Order of the auditory stimulus (SOV: *M* = 85.9%, *SD* = 13%, and OSV: *M* = 84.4%, *SD* = 13.8%).

In order to estimate the effect of Session and Pretraining on lexical learning, data were submitted to a mixed-effects logistic regression model. The model had Accuracy as categorical dependent variable and Session (contrast coded as -1.5, -1, -0.5, 0.5, 1 and 1.5 for Sessions 1 to 6, respectively), Word Order (contrast coded with OSV as -0.5 and SOV as 0.5), Pretraining and Metalinguistic Awareness, both scaled and centered, and all the two-way interactions as predictors. The procedure for building the model and for identifying the best random-effects structure was identical to that described in Study 1. The model had intercepts for participants and items, by-participant random slopes for Session, Word Order, and by-item random slopes for Session.

**Fig 3:**
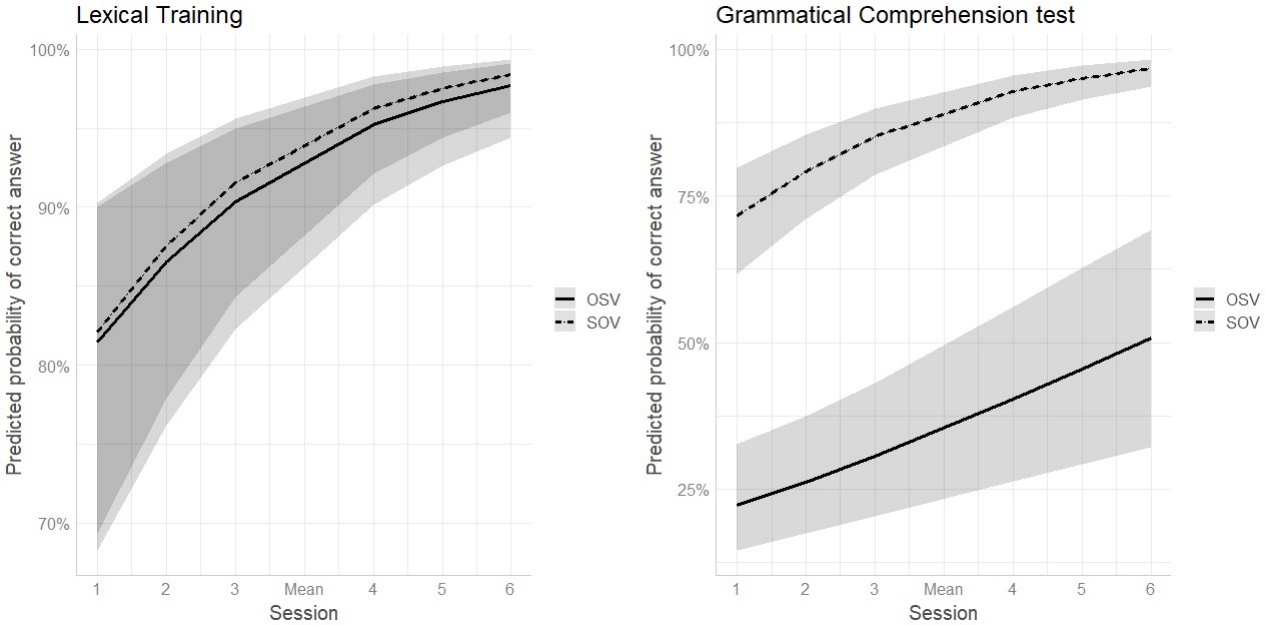
Predicted probability of a correct answer as a function of Session and Word Order in Lexical Training (left) and Grammatical Comprehension test (right) in L1 German participants.

The results, shown in Table 10 revealed main effects for Session and Word Order which indicates that response accuracy improved for both word orders across sessions and was higher for SOV than for OSV sentences, as can be seen by the positive coefficients of the effects and the predicted probabilities for the two word orders (95% and 92% respectively). A significant interaction between Session and Word Order was also found. Post-hoc analyses revealed that the effect of Session was stronger for SOV sentences (Fig 3), suggesting that, as the study progressed, participants were more likely to select the correct image upon hearing sentences with the SOV structure as compared to when hearing OSV sentences. Furthermore, there were significant effects of Pretraining and Metalinguistic Awareness suggesting that participants with better scores on these measures exhibited greater lexical learning gains. The model also showed a significant interaction between Metalinguistic awareness and Session, for which a follow-up simple slope analysis showed that the effect of Metalinguistic Awareness varied across sessions, becoming stronger over time.

**Table 10:** Mixed-effects model fitted to the lexical training data from L1 German participants.

Variable	*B*	*SE*	*Z*	*P*	*Odds Ratios (CI)*
Intercept	2.714	0.370	7.329	< .001	15.09 (7.30–31.18)
Session	0.808	0.129	6.265	< .001	2.24 (1.74–2.89)
Word Order	0.200	0.071	2.830	.005	1.22 (1.06–1.40)
Pretraining	0.670	0.209	3.203	.001	1.95 (1.30–2.94)
Metalinguistic Awareness	0.800	0.207	3.869	< .001	2.23 (1.48–3.34)
Session: Pretraining	0.105	0.099	1.063	.288	1.11 (0.92–1.35)
Session: Word Order	0.106	0.037	2.875	.004	1.11 (1.03–1.20)
Session: Metalinguistic Awareness	0.273	0.098	2.773	.006	1.31 (1.08–1.59)
Word Order: Pretraining	0.068	0.044	1.535	.125	1.07 (0.98–1.17)
Word Order: Metalinguistic Awareness	-0.001	0.047	-0.010	.992	1.00 (0.91–1.10)
Pretraining: Metalinguistic Awareness	0.222	0.158	1.403	.161	1.25 (0.92–1.70)
*Random effects*	Variance	SD			
Item: Distractor Type (intercept)	0.132	0.364			
Participant (intercept)	1.401	1.184			
Distractor Type (intercept)	0.291	0.540			
Item: Distractor Type | Session (slope)	0.025	0.159			
Participant | Session (slope)	0.293	0.542			
Participant | Word Order (slope)	0.021	0.145			
Distractor Type | Session (slope)	0.024	0.155			
Marginal R^2^	.300				
Conditional R^2^	.584				

#### Grammatical comprehension test

As shown in Table 8, participants, on average, demonstrated above chance performance across all sessions. Learners’ ability to correctly identify the target scene seemed to improve between Sessions 1 and 2 and Session 2 and 3. However, accuracy remained stable from this point onwards, albeit with small fluctuations. In terms of Word Order, participants were more accurate with SOV (*M* = 81.7%, *SD* = 15%) than with OSV sentences (*M* = 40.1%, *SD* = 28.6%).

In order to determine whether there were any associations between participants’ reported level of explicit knowledge of the target grammatical rules and grammatical comprehension, a mixed-effects logistic regression model was used. Accuracy was modeled as a binary dependent variable. Metalinguistic awareness and Pretraining, Word Order and Session, both contrast coded as in the model on Lexical Training, and all the two-way interactions were added as fixed effects. The random-effects structure included random intercepts for participants and items as well as by-participant random slopes for Session, Word Order and Metalinguistic Awareness and a by-item random slope for Pretraining. The model building procedure was identical to that detailed in Study 1.

The results of the model are shown in Table 11. The model returned significant main effects for Session and Word Order, which means that while learners improved significantly across sessions, accuracy throughout the study was driven primarily by correct responses on SOV sentences, which corresponds to an estimated 90% accuracy as opposed to 35% for OSV sentences. Additionally, there was a significant interaction between the two predictors which was followed up by post hoc analysis that revealed that the difference in performance on SOV and OSV stimuli remained significant across sessions (Fig 3). The model also showed a significant effect of Metalinguistic Awareness indicating that learners’ level of conscious knowledge was predictive of grammatical comprehension scores. Moreover, there was a significant interaction between Session and Metalinguistic Awareness. A post-hoc simple slope analysis revealed that the effect of Metalinguistic Awareness became progressively more pronounced over the course of the study. In the first session, it was negative and nonsignificant. In the second session, it became positive but remained nonsignificant. It finally reached significance in Session 3 and was significant in all the subsequent sessions.

**Table 11:** Mixed-effects model fitted to the grammatical comprehension test data from L1 German participants.

Variable	*B*	*SE*	*Z*	*P*	*Odds Ratios (CI)*
(Intercept)	0.770	0.209	3.675	< .001	2.16 (1.43–3.26)
Word Order	2.753	0.339	8.118	< .001	15.68 (8.07–30.48)
Session	0.620	0.107	5.815	< .001	1.86 (1.51–2.29)
Pretraining	0.132	0.148	0.891	.373	1.14 (0.85–1.53)
Metalinguistic Awareness	1.058	0.283	3.734	< .001	2.88 (1.65–5.02)
Word Order: Session	0.387	0.046	8.458	< .001	1.47 (1.35–1.61)
Word Order: Pretraining	-0.088	0.345	-0.255	.799	0.92 (0.47–1.80)
Session: Pretraining	0.056	0.114	0.488	.626	1.06 (0.85–1.32)
Session: Metalinguistic Awareness	0.745	0.135	5.540	< .001	2.11 (1.62–2.74)
Word Order: Metalinguistic Awareness	-0.028	0.384	-0.074	.941	0.97 (0.46–2.06)
Pretraining: Metalinguistic Awareness	0.318	0.174	1.827	.068	1.37 (0.98–1.93)
*Random effects*	Variance	SD			
Item (intercept)	0.173	0.416			
Participant (intercept)	0.562	0.750			
Item | Pretraining (slope)	0.015	0.123			
Participant | Word Order (slope)	3.788	1.946			
Participant | Session (slope)	0.370	0.608			
Participant | Metalinguistic Awareness (slope)	0.483	0.695			
Marginal R^2^	.406				
Conditional R^2^	.654				

#### Grammaticality judgment tasks

Table 12 displays the descriptive statistics for accuracy scores in the two GJTs. Overall, in both tasks, performance at the group level was above chance (for GJT 1: *M* = 71.3%, *SD* = 12.2%, and for GJT 2: *M* = 72.4%, *SD* = 13.1%). Participants exhibited similar performance patterns in both tasks, with better performance on grammatical than ungrammatical sentences and on sentences containing word order violations (Verb placement and Adjective before noun) than on sentences with case marking errors (Adjective noun agreement, Two accusatives and Two nominatives).

**Table 12:** Mean percentage correct responses (SDs) and d’ scores by sentence type the grammaticality judgement task for the L1 German learners.

Condition	GJT 1 (Session 5)			GJT 2 (Session 6)		
	Grammatical	Ungrammatical	d’	Grammatical	Ungrammatical	d’
	M (SD)	M (SD)	M (SD)	M (SD)	M (SD)	M (SD)
*Word order violations*						
Verb placement	95.1 (7.4)	96.4 (9.2)	2.80 (0.45)	93.1 (14.2)	99.0 (4.5)	2.87 (0.51)
Adjective placement	87.5 (18.6)	60.9 (25.2)	1.44 (0.89)	90.1 (13.4)	70.1 (23.7)	1.79 (0.85)
*Case marking violations*						
Nominative marking	93.1 (10.4)	29.3 (35.6)	0.67 (1.09)	94.4 (10.4)	24.0 (36.5)	0.52 (1.16)
Accusative marking	97.0 (6.1)	26.0 (36.3)	0.70 (1.16)	98.0 (6.8)	25.7 (37.1)	0.78 (1.16)
Adjective noun agreement	91.5 (13.0)	35.9 (36.2)	0.83 (1.16)	90.1 (13.7)	39.8 (39.5)	0.86 (1.16)
Overall	92.8 (7.9)	49.7 (23.4)	1.63 (0.93)	93.2 (10.5)	51.7 (24.8)	1.79 (0.97)

Data from the two GJTs were analyzed jointly in a mixed-effects logistic regression model. The model included Accuracy as the outcome variable and Metalinguistic Awareness, Pretraining, Word Order (all coded as in the Grammatical Comprehension test model above), Grammaticality (contrast coded with ungrammatical sentences as -0.5, and grammatical as 0.5), Error Type (contrast coded with case marking sentences as -0.5 and word order sentences as 0.5), Test Time (contrast coded with GJTTime 1 as -0.5, and GJTTime2 as 0.5) as predictors. The model’s random-effects structure included random intercepts for participants and items, by-participant random slopes for Grammaticality and Error Type and by-item random slopes for Test Time and Metalinguistic Awareness.

**Fig 4:**
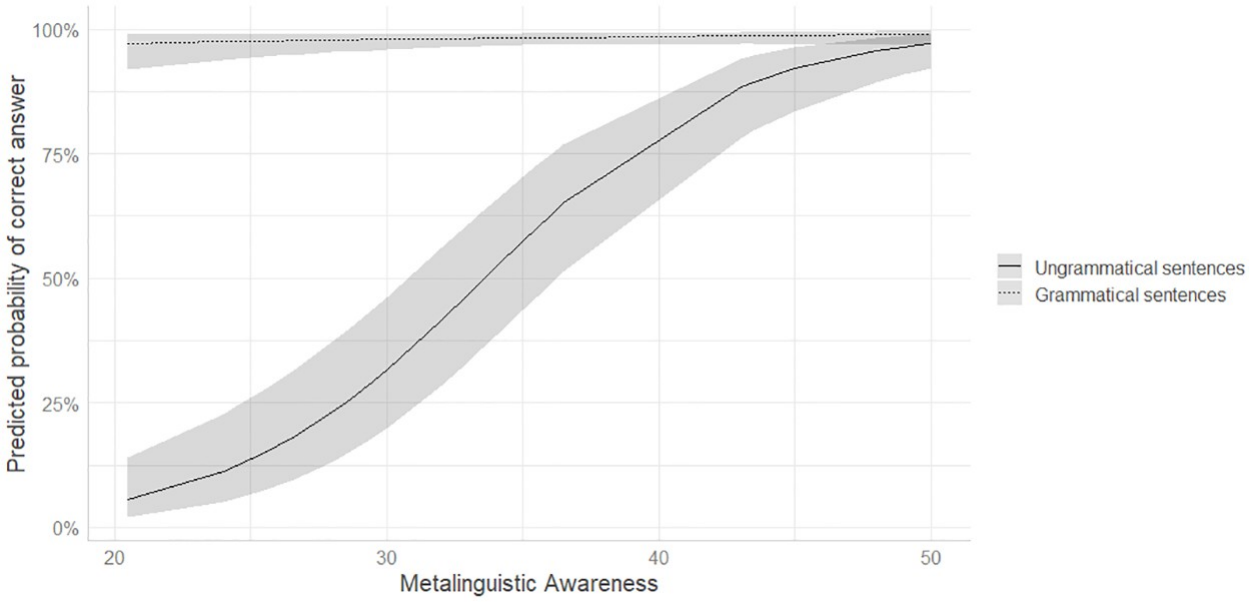
Predicted probability of a correct answer as a function of grammaticality and metalinguistic awareness scores in the grammaticality judgement task.

The model output (See S4 Appendix for the full model) showed significant effects of Grammaticality (*β* = 3.226, *z* = 6.695, *p* < .001) and Metalinguistic Awareness (*β* = 1.02, *z* = 6.725, *p* < .001), indicating that participants were less accurate when judging ungrammatical items relative to grammatical ones, with a difference in the predicted probability of a correct answer of 30% (68% vs. 98%), and that participants with higher scores on the metalinguistic awareness questionnaire were more likely to judge the grammaticality of the sentences correctly. In addition, the model returned significant interactions between Grammaticality and Pretraining (*β* = 0.878, *z* = 2.062, *p* = .039) and between Grammaticality and Metalinguistic Awareness (*β* = -1.507, *z* = -3.551, *p* < .001). Follow-up simple slope analyses showed that the effect of Pretraining was significant only for grammatical sentences, whereas the effect of Metalinguistic Awareness was significant only for ungrammatical sentences (Fig 4). A significant positive coefficient for Error Type (*β* = 2.206, *z* = 7.428, *p* < .001) was also detected, meaning that, overall, learners were more accurate in judging the grammaticality of word order than case marking sentences (97% and 78% predicted accuracies respectively). Moreover, the model showed significant interactions between Error Type and Grammaticality (*β* = -5.020, *z* = -8.783, *p* < .001) and between Error Type and Pretraining (*β* = 0.400, *z* = 2.600, *p* = .009). With regard to the Error Type and Grammaticality interaction, the result of follow-up analyses revealed that the effect of Grammaticality was stronger and significant only for sentences in the case marking condition. The probability of a correct answer was higher for grammatical (98%) than for ungrammatical sentences (17%), while no such difference was observed for word order sentences (98% vs. 96%). With respect to the interaction between Error Type and Pretraining, a simple slope analysis showed that the effect of Pretraining was significant only for word order sentences. Last, an interaction between Test Time and Awareness emerged, and the results of a simple slope analysis showed that the effect of Test Time was stronger for participants who achieved higher scores on the metalinguistic awareness questionnaire and significant only for those with higher scores (mean +1SD).

#### Final grammatical comprehension tests

Descriptive statistics of the scores on the FGCTs in Session 5 (FGCT 1) and Session 6 (FGCT 2) revealed that learners were more accurate with SOV (for FGCT 1: *M* = 83.2%, *SD* = 18.3%, and for FGCT 2: *M* = 86.5%, *SD* = 16.1%) than OSV items (for FGCT 1: *M* = 44%, *SD* = 36.3%, for FGCT 2: *M* = 44.7%, *SD* = 40.2%).

Results from the two tests were analyzed using a mixed-effects logistic regression model. Accuracy was used as the outcome variable and Metalinguistic Awareness, Pretraining, Word Order and Test Time (contrast coded with FGCT 1 as -0.5 and FGCT 2 as 0.5) and all their two-way interactions were included as predictors. The best-fitting model contained random intercepts for participants and items, by-participant random slopes for Word Order.

The model (See S4 Appendix for the full model) revealed main effects of Word Order (*β* = 2.865, *z* = 5.192, *p* < .001), Test Time (*β* = 0.377 *z* = 2.715, *p* = .007) and Metalinguistic Awareness (*β* = 1.960, *z* = 7.262, *p* < .001). The positive estimates for the effects suggest that participants performed significantly better on SOV compared to OSV items (estimated accuracies of 94% and 45% respectively), that accuracy improved significantly between FGCT 1 and 2 (estimated accuracies of 74% and 81% respectively), and that participants with higher Metalinguistic Awareness scores showed better grammatical comprehension skills. Finally, there were interactions, though not significant, between Pretraining and Metalinguistic Awareness (*β* = 0.557, *z* = 1.904, *p* = .057) and between Test time and Metalinguistic Awareness (*β* = 0.359, *z* = 1.882, *p* = .060). Simple slope analyses revealed that the effect of Metalinguistic Awareness was less strong for participants with lower Pretraining scores and stronger for FGCT 2 than for FGCT 1.

#### L1 experience effects

To investigate the impact of prior L1 experience on L2 grammatical learning, the L1 German group’s performance on the GJT1 and the FGCT1, both administered in session 5, was compared to that of the L1 English learners from Study 1. Before completing these tasks, both groups had received the same amount of exposure to the artificial language.

We first analyzed data from the GJT using a mixed-effects logistic regression model, with Accuracy (correct = 1, incorrect = 0) modeled as the binary outcome variable. The model included Grammaticality (contrast coded as in the GJT model above), Group (contrast coded, with the L1 English group as -0.5 and the L1 German group as 0.5), Error Type (contrast coded with case marking sentences as -0.5 and word order sentences as 0.5) and their interaction as predictors. The random-effects structure of the model had random intercepts for Participants and Items, and random slopes for Grammaticality and Error Type over Participants and random slopes for Group over Items.

**Fig 5:**
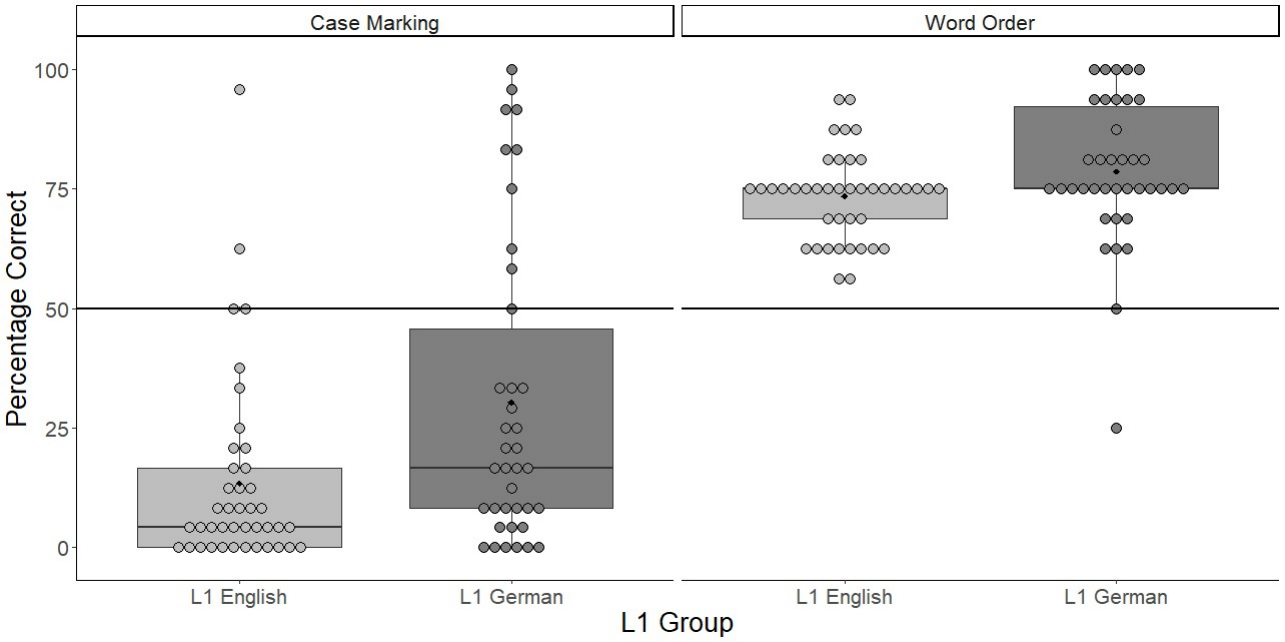
The distribution of scores across L1 groups and types of violation. The black diamonds indicate group means and the solid blacklines indicate chance accuracy level.

The model (See S4 Appendix for the full model) revealed main effects of Grammaticality (*β* = 3.731, *z* = 7.806, *p* < .001) and Error Type (*β* = 2.395, *z* = 6.603, *p* < .001), indicating that participants in both groups performed better on grammatical than ungrammatical sentences and on word order than case marking sentences. Furthermore, a significant interaction between Grammaticality and Error Type (*β* = -4.159, *z* = -6.124, *p* < .001) was detected, for which post-hoc analysis showed that, overall, the effect of Grammaticality was significant for both error types but stronger for items in the case marking condition. Furthermore, the interaction between Group and Error Type (*β* = -0.792, *z* = -2.339, *p* = .019) was also significant, and a post-hoc analysis revealed that the L1 German learners performed significantly better on case marking sentences compared to their L1 English counterparts (with an estimated 76% accuracy vs. 59% respectively). This difference in performance was particularly evident in learners’ judgements of ungrammatical sentences containing case marking violations (Fig 5).

Finally, another mixed-effects logistic regression model was built to determine the potential effect of L1 on groups’ performance in the first FGCT. Accuracy was entered as the dependent variable and Word Order (contrast-coded), Group (contrast-coded as in the previous two models) and their interaction as predictors. The model (See S4 Appendix for the full model) also had random intercepts for participants and items, and by-participant random slopes for Word Order. The model returned significant effects for both Word Order (*β* = 2.632, *z* = 6.883, *p* < .001) and Group (*β* = 0.783, *z* = 2.645, *p =* .008), showing that, while both groups performed better on SOV than on OSV sentences, the L1 German had higher accuracy overall compared to the L1 English group (with an estimated 74% accuracy vs. 55% respectively).

### Discussion

The primary goal of Experiment 2 was to test whether the results of Experiment 1 would be replicated with learners who have a different L1 background. In line with our prediction, L1-German learners were found to be more accurate than the L1-English group in all grammar tasks. This difference in performance appears to be consistent with previous studies suggesting that L2 sentence processing is modulated by learners’ L1 background, such that syntactic structures that are similar in L1 and L2 are learned more easily (e.g., [102, 107]), reflecting positive transfer from L1 to a new language in the absence of instruction on grammar structure.

Despite the differences in grammatical comprehension abilities between the two groups, closer inspection of the data revealed compelling similarities in their learning outcomes. Specifically, both groups had higher accuracy rates on SOV than on OSV sentences and were more accurate on judging sentences as ungrammatical when they contained a word order violation as opposed to when they involved a case marking error. These findings suggest that, despite experiencing positive transfer effects, adults continue to face difficulties in learning new grammatical rules under incidental conditions.

Another contribution of Experiment 2 involved testing whether the provision of additional incidental exposure suffices for obtaining stronger learning effects. Results demonstrated that increasing exposure, by providing 540 additional lexical training items over 2 sessions, leads only to very small learning gains. Performance in the Grammatical Comprehension blocks increased by 2.5% from session 4 to session 6 (i.e., ~2 additional correct responses over the last 2 sessions), while overall accuracy increased only marginally between GJT1 and GJT2. Counter to these results, learners became significantly more accurate from FGCT1 to FGCT2. However, this increase in accuracy came only from better identification of SOV sentences. Collectively, while Experiment 2 does not allow for a clear answer to the question of whether further incidental exposure can lead to better grammatical comprehension skills–the possibility that one or two additional sessions of exposure could result in significant increases in accuracy, albeit unlikely, cannot be precluded–, it shows the inefficiency of incidental learning as the sole L2 learning mode.

Finally, the data suggest a strong link between the development of grammatical comprehension, even under incidental conditions, and the emergence of conscious knowledge about the target linguistic structures. Characteristically, although performance in the Grammatical Comprehension blocks improved over time, this improvement was detected only in participants who exhibited average or above average performance in the post-test questionnaire. What’s more, the metalinguistic awareness effect became stronger over time. While counterintuitive at first sight, this finding seems to reflect the fact that learners likely required a relatively large amount of input before developing an understanding of the target grammatical structures. As shown in Table 8, participants showed limited grammar learning in session 1 (55.5%), which may have not been enough for such an effect to emerge. However, as performance began to diverge from chance and learning scores improved, the effect of metalinguistic awareness became more pronounced. Learners with high metalinguistic awareness scores were also more likely to perform better in both GJT and FGCT. Regarding the former task, metalinguistic awareness was found to be more predictive of participants’ accuracy in ungrammatical sentences, with aligns with earlier findings that responses to this type of stimuli are more likely to tap into learners’ explicit knowledge [108]. Analyses of the data also revealed a relationship between degree of metalinguistic awareness and lexical learning. First, participants who demonstrated a higher level of awareness of their grammatical knowledge performed better in the Lexical Training task. Secondly, in both the Grammatical Comprehension test and the FGCT, the effect of metalinguistic awareness was greater for learners who achieved higher scores in the Pretraining task. Overall, these findings appear to highlight the facilitative role of explicit knowledge that is available to awareness in the process of learning new grammatical structures [106, 109].

## General discussion

The current study examined adult learners’ capacity to learn new grammatical rules from incidental exposure. Our main goal was to examine whether adults can learn different aspects of grammar incidentally and the degree to which learning is modulated by their prior L1 experience. In the two experiments presented here, learners with different L1 backgrounds (English and German) were exposed to Kepidalo, a new artificial language that included case marking while allowing for a relatively flexible word order with a canonical SOV and a non-canonical OSV order. Furthermore, in an effort to extend previous literature, we investigated if extensive incidental exposure can lead to higher levels of learning than those reported in previous studies and whether the degree of learners’ conscious knowledge of the grammatical regularities contributed to their learning outcomes.

### Learning novel word order and case marking rules through extensive incidental exposure (RQ1, RQ2, & RQ3)

The results of Experiments 1 and 2 showed that, even after extensive incidental exposure to the artificial language, overall, both L1-English and L1-German learners continue to exhibit only partial knowledge of the language’s grammatical rules. These findings appear to align well with previous artificial language studies showing that learning nonnative structures under incidental conditions poses a great challenge for adult learners, leading to generally low learning effects (e.g., [11, 13, 40]).

That is, however, not to say that the learning difficulties observed apply uniformly to all grammatical aspects of the language. Rather, it appears that participants succeed in learning certain aspects of word order. Evidence of this comes from the GJT, where performance on sentences containing verb placement violations was found to be close to ceiling, indicating that participants from both language groups possessed well-developed knowledge of the verb-final rule of the language. This is in accord with the learning outcomes reported by Rebuschat et al. [12] and Walker, Monaghan, Schoetensack, and Rebuschat [110]. We hypothesize that the high accuracy on trials involving verb placement violations observed here can be attributed to a number of factors that work in unison. Firstly, the rigidity of verb placement may have rendered the verb position a consistent and reliable cue to word order, making it more accessible and learnable to participants. Secondly, given that items that appear towards the end of sentences are privileged during sentence processing [111–113], it is likely that the systematic occurrence of verbs in sentence-final position may have increased their overall perceptual salience, making it easier for participants to learn them and, consequently, identify them in the sentence. This allowed learners to notice the erroneous verb placements in the GJT and correctly judged these sentences as ungrammatical. A third factor may relate to the morphological properties of the novel verbs. In fact, all nonce-verbs created for this study were phonologically similar. They all were disyllabic, had the same syllable structure (CVCVC) and carried the suffix -ek. These properties may have additionally increased the salience of words referring to verbs and facilitated their recognition. Thus, to detect the ungrammaticality of the sentences, participants may have simply relied on the absence of these target properties from the sentence-final word. Finally, learners’ knowledge of the verb placement rule may be tightly linked to the effective learning of verb referents [12, 110]. This seems to be fully in line with previous research demonstrating robust relationship between the development of vocabulary and grammar (e.g., [12, 114–116]).

The GJT results also suggest that, by session 5, participants had developed an at least partial understanding of the noun–adjective word order. However, the learning effects observed were not equivalent for the two language groups. While the L1 German participants responded with above-chance accuracy on sentences containing adjective placement violations (Table 12), the L1 English learners’ performance was not greater than chance (Table 7). Following the above-mentioned claim regarding the relation between vocabulary and grammar, we argue that one likely reason for the difference in accuracy between the two groups may be tied to the better learning of the word-referent mappings for adjectives in the L1 German group (*M* = 78.1%) compared to the L1 English group (*M* = 68.9%). A second reason for these results may stem from participants’ previous foreign language learning experiences. While none of the L1 German learners had previous knowledge of additional case marked languages, all of them had learned English and nearly all of them (33/38) had been introduced to either Spanish or French in the school setting (foreign language learning is compulsory in Germany). Importantly, in both Spanish and French, color adjectives strongly tend to occur in post-nominal position [117, 118]. Although none of the German participants included in this study reported working knowledge of any additional languages other than English, it remains possible that their earlier linguistic experience with Spanish or French may have facilitated the detection of similarities between the previously taught languages and Kepidalo, leading to positive transfer effects. Such a possibility would be in line with the idea of lexical and syntactic transfer from L2 to L3 [119–121].

Yet, the learning of the noun–adjective word order was not as effective as that observed for the verb placement rule. We hypothesize that this difference in learning outcomes is directly related to input frequency. Indeed, there is a great deal of research emphasizing the role of frequency in L2 learning [52, 122–124]. Similar to L1 acquirers, L2 learners are sensitive to the frequency of syntactic constructions in the input, and thus, constructions that occur frequently are often more fluently processed than the less frequent ones. In Kepidalo, in contrast to nouns and verbs, adjectives were optional and appeared only in half of the sentences, providing fewer opportunities for learning. This is reflected in the lower learning outcomes for both the referents for adjectives and the noun-adjective word order, compared to the referents for verbs and the verb placement rule. Further support for the role of input frequency comes from the finding that additional exposure to language led to an improvement in L1 German participants’ performance on adjective placement violation trials between sessions 5 and 6.

Finally, further evidence of grammar learning emerges from participants’ responses in the Grammatical Comprehension tests. In particular, throughout the study, participants in both language groups exhibited superior performance on sentences that had the canonical SOV word over those with the non-canonical OSV sentences. This pattern of performance may be linked to the relative frequencies of occurrence of the two word orders in the input. Hence, it is likely that the higher frequency of the SOV word order promoted learning of this pattern. An additional factor that may have contributed to this frequency effect can be traced to the noun pretraining task. Recall that in that task, all nouns appeared in the nominative case only (e.g., Alg-i). Thus, the early presentation of nouns in the nominative case ending might have led participants to memorize these forms as unanalyzable chunks (e.g., Algi) and take them to be the only potential word-forms that can be mapped onto the different referents. Later, the increased likelihood of occurrence of these word-forms in the highly salient sentence-initial position during the lexical training and grammar test blocks may have facilitated their recognition, resulting in higher accuracy on the SOV sentences.

The learning of the canonical SOV pattern can also be taken to reflect a learners’ subject-first preference when assigning grammatical roles to noun phrases in transitive sentences [125–127]. One potential explanation for this preference is that placing the subject/agent before the object/patient allows learners to engage a simple sequential processing strategy for determining the meaning of the sentences. Given the increased complexity of the artificial language, and due to lack of explicit instruction, learners may have adopted this strategy as it is less cognitively taxing than using case marking information [128, 129]. Accordingly, the stronger subject-first preference observed in the initial stages of exposure may reflect learners’ tendency to adopt this strategy, firstly, due to insufficient evidence regarding the presence of a second word order and, secondly, because it frees up more cognitive resources which they can use for processing the sentences for meaning, given that they are still attempting to learn the novel vocabulary.

A further explanation for the better learning attested for the SOV pattern can be linked to L1 experience effects. However, the manner in which these effects emerge are different for the two language groups. First, regarding the L1 German participants, L1 effects may stem directly from the application of L1-based strategies on L2 sentence processing. Native speakers of German tend to exhibit a preference for subject-first (SOV) readings when presented with Noun-Noun-Verb sentences in their L1 [130–132]. Accordingly, similar to our findings, it has been shown that L1 German L2 learners are also inclined to interpret non-native Noun-Noun-Verb strings as SOV [133–135]. On the other hand, the effect of L1 experience follows a more indirect path in the case of the L1 English learners. In contrast to native speakers of German, L1 English speakers usually assign an OSV interpretation to Noun-Noun-Verb sequences in their native language [133, 136]. Thus, the pattern of performance observed here cannot be directly attributed to L1 transfer. Rather, our results appear to be in line with the idea of meta-transfer [137]. In order to determine the grammatical roles of subject and object in a sentence, native speakers of English rely strictly on word order cues. Subsequently, when exposed to a new language, instead of simply employing the English surface word order (i.e., SVO), L1 English learners tend to transfer their sensitivity to word order as the main processing strategy from their L1 to L2. Thus, potential meta-transfer effects are thought to be strongly constrained by input frequency. In this study, the higher frequency of the SOV order as well as the early presentation of nouns in the nominative case may have led participants to abandon their L1-based preferences for SVO, or for OSV, when presented with Noun-Noun-Verb sentences, in favor of the interpretation that was more available, namely SOV. Interestingly, our results seem to corroborate earlier findings from natural language learning studies showing that, in contrast to native speakers of Japanese, L1 English learners of Japanese tend to over-rely on the canonical SOV order [137–139].

Despite our participants’ success with the canonical SOV order, accuracy on OSV sentences in both groups was below chance levels across all sessions in both groups. While factors like input frequency and L1 experience can be used to account for the better learning of the canonical order documented in Experiments 1 and 2, they can equally be employed for explaining participants’ difficulties with the non-canonical OSV order. Indeed, previous studies have shown that grammatical constructions that occur less frequently in the input or share less structural properties with previously learned languages present more processing difficulties for non-native speakers and tend to be acquired later [140–143].

Most importantly, however, the limited learning of the non-canonical word order could be seen as an outcome of participants’ struggles with learning case marking. Results of the GJT in both Experiments 1 and 2 revealed that participants showed a strong tendency to incorrectly accept ungrammatical trials that contained case marking violations (Tables 7 & 11). This appears to be fully in agreement with the well-documented difficulties of adult learners in processing and acquiring L2 inflectional morphology (e.g., [51, 60, 144, 145]).

One of the main sources of these difficulties is thought to be the low perceptual salience of inflectional morphemes [146, 147]. In contrast to most lexical items, morphemes are usually made up of a single segment or syllable, are unstressed, and, due to their word-final position, are often likely to be fused with surrounding items, making them hard to perceive and learn. These low salience effects can be additionally influenced by two factors, namely learned attention and decomposition. The fact that neither English nor German mark the singular nominative and accusative cases on nouns, may have led learners to direct their attention to other L1-related cues for interpretation, instead of attending to case marking. While word order appears to be an obvious candidate for native speakers of English, as mentioned earlier, we suspect that this was also the cue L1 German learners use, at least during the initial stages of learning. Secondly, limitations in adult learners’ decomposition ability [148, 149], may have led participants to treat the novel words as unanalyzable wholes, instead of decomposing them into stems and suffixes, hampering the detection and processing of case marking information. It should, however, be mentioned that, although both language groups experience difficulties in learning case marking, given the marginal use of case marking in English, these are far more pronounced in the L1 English group. In sum, these results seem to further confirm the idea that acquiring L2 morphology incidentally can be particularly challenging for adult learners [11–13, 40], while also demonstrating that such learning difficulties can persist even for learners who have native knowledge of a morphologically rich language.

In light of these findings, the present study joins the handful of previous studies examining the simultaneous learning of word order and case marking [11, 12, 110] in showing that word order is more susceptible to learning in incidental contexts of exposure and, as a result, acquired faster. Importantly, while learned attention and cue salience tend to reduce the noticeability of grammatical morphemes rendering them less learnable for non-native speakers, sensitivity to sequential probabilities is thought to remain available in adults [16, 150], enabling them to extract sequential patterns from structured or unstructured input. Indeed, a number of studies have found a strong association between sequential learning and L2 sentence processing (e.g., [151, 152]). In fact, sensitivity to sequential/temporal cues appears to be pervasive in L2 acquisition. For instance, work on cue weighting in L2 speech perception shows that early L2 learners tend to rely primarily on temporal (i.e., duration) instead of spectral information in order to distinguish between speech sounds [153–155], even in cases where their L1 makes little use of duration [156]. Thus, overall, our findings appear to highlight the important role of sequential processing in L2 acquisition [8, 157, 158].

Another objective of the present study was to examine whether the provision of extensive incidental exposure to input can increase the robustness of novel grammar learning (RQ3). Our results show that despite the improvement in participants’ performance over the course of the study, not all aspects of grammar were learned reliably. Specifically, while extensive incidental exposure was sufficient for learning rules related to surface word order patterns, it was not enough to lead to the acquisition of case marking. Thus, our results echo previous studies evidencing relatively low learning effects for L2 case marking under incidental conditions (e.g., [11, 13]), thereby suggesting that in order for low salient morphosyntactic forms such as inflectional morphemes to be effectively learned by adult L2 learners, explicit types of instruction, as well as sufficient exposure, should be considered basic preconditions [4, 159, 160]. It should, however, be acknowledged that although the training regimen employed here is described as extensive, the amount of artificial language input provided makes up only a limited proportion of the input learners usually receive in naturalistic contexts over an extended period of time. Hence, the observed learning outcomes can only be taken to be reflective of the very early phases of language acquisition.

### The effect of L1 experience on incidental L2 grammar learning (RQ4)

Turning to the fourth research question, our results indicate the presence of significant L1 effects in the learning of novel grammatical constructions. Specifically, we found that the L1 German learners demonstrated better grammatical comprehension abilities than their L1 English counterparts and were more sensitive to both case marking and word order violations. Characteristically, the number of participants who demonstrated learning of case marking was highest among the L1 German group (Fig 4). In addition, according to the results from the Grammatical Comprehension blocks the discrepancy in performance between the two language groups seems to be numerically greater in the later sessions, indicating that the influence of L1 may become stronger as participants receive more input. Thus, although providing extensive exposure to input under incidental conditions did not result in robust grammar learning, it allowed for strong L1 effects to emerge.

These findings, however, do not mean that L1 experience does not play a role upon the very first exposure to the novel language. In fact, as mentioned earlier, such effects are expected to be at play from the very first stages of L2 learning [161, 162] influencing learners’ preference for certain cues over others (e.g., SOV over OSV or word order over case marking). What could, then, cause this delayed emergence of the influence of L1 background in the accuracy rates of the two language groups? Firstly, early L1 effects may have been masked by the increased complexity of the Grammatical Comprehension test in the initial stages of exposure. In addition, the meaning-focused nature of the preceding Lexical Training task may have biased participants towards processing the language for meaning, leading learners from both groups to engage equivalent processing strategies and, hence, hindering the detection of L1-specific influence. Most importantly, this pattern of development appears to be congruent with earlier research suggesting a piecemeal integration of cues during the early stages of L2 learning [22, 62, 163–165]. In particular, learners are thought to first rely on a single cue or pattern and upon learning it, they proceed to the next one, which, in turn, enables them to use both cues in a combinatory manner. The order in which different cues are learned is largely influenced by their availability (i.e., how often they are present in the L2 input) and by transfer effects stemming from prior L1 knowledge. Therefore, the developmental pattern that emerged from the current study can be seen to reflect this incremental learning process. During the initial stages of exposure, learners from both L1 groups relied on the more salient and available SOV order as the main cue to agent and patient identification. As they received more input, their performance began to diverge. While the vast majority of L1 English learners continued to show a strong reliance to word order, given its dominance in L1 processing, L1 German learners were more likely to demonstrate a gradual shift from the initial reliance to word order to the more reliable case marking, benefitting from their prior extensive experience with case marking. Despite this, it should be noted that although the aforementioned developmental pattern accounts well for the current data, learners’ performance was subject to large individual differences. Indeed, not all native speakers of German exhibited learning of case marking and, conversely not all participants from the L1 English group failed to pick up on the case marking cue. All in all, the results show that prior L1 experience plays a crucial role in learning novel grammatical constructions under incidental exposure conditions. However, a considerable amount of input exposure may be required in order to capture such L1 effects.

### The role of awareness in incidental L2 grammar learning (RQ5)

Our final research question concerned the extent to which learning grammar under incidental conditions is contingent upon the development of explicit knowledge of which learners are aware. As already stated in the interim discussion, the effect of metalinguistic awareness was ubiquitous across all tasks administered, underscoring its role in the course of the L2 learning process and its importance for the less salient aspects of grammar [1, 160, 166–168]. Crucially, this indicates that the emergence of metalinguistic awareness of the underlying grammatical patterns remains predictive of learning outcomes even in conditions where learning occurs unintentionally and without explicit feedback.

Overall, the findings yielded by this study raise two important points that are worth discussing. First, the present results are compatible with previous findings from artificial language learning studies showing that higher levels of awareness, either at the level of noticing or at the level of understanding [106], are associated with greater learning gains following incidental exposure, a trend that appears to be particularly strong for learning of case marking (e.g., [11, 13, 17, 40]). Furthermore, although our participants’ level of awareness was identified as a strong predictor of grammar learning, its effect only reached significance by the third session, indicating that a substantial amount of incidental exposure is likely required before making valid explicit inferences about the less salient grammatical aspects of a novel language.

Secondly, and building on the previous point, even though the predictive power of conscious awareness suggests that L2 grammar learning was largely driven by explicit knowledge, it does not mean that implicit knowledge was entirely absent. Note that we relied on offline measures to gauge both participants’ level of metalinguistic awareness and learning outcomes. Awareness was assessed via a retrospective questionnaire in which, similar to retrospective verbal reports, the provision of a correct answer or rule description does not necessarily entail that learners have only developed explicit knowledge [39, 169]. Grammar learning, on the other hand, was assessed using untimed 2AFC tasks and GJTs, both of which are thought to allow participants to consciously reflect upon the learned material before arriving to an answer, increasing the likelihood of engaging metalinguistic processes [160]. Thus, it is possible that the effect of metalinguistic awareness as well as the learning effects may be somewhat modulated by task-specific characteristics. The extent to which similar results can be obtained using online measures of learning (e.g., visual-world eye-tracking paradigm, word-monitoring task) is a question that needs to be further explored (see Pili-Moss [81] for similar results with a different type of accuracy measure). Nevertheless, the present findings appear to be in line with earlier research on L2 learning suggesting that the emergence of metalinguistic awareness plays an important, if not critical, role for the creation of L2 grammatical knowledge [1, 166, 168].

## Conclusion

Using a novel artificial language, Kepidalo, the current study attempted to provide further insight into adult learners’ ability to acquire new grammatical structures under incidental exposure conditions. While more replications are required in order to verify the generalizability of the reported results, the current set of findings replicates and extends earlier studies by demonstrating that learning difficulties, particularly with the less salient aspects of grammar, persist even after extensive exposure to language input, indicating that incidental language learning continues to pose a significant challenge for adult learners. Nevertheless, the magnitude of the difficulties was found to differ as a function of L1 background. In particular, leveraging their prior experience with a morphologically rich L1, native speakers of German showed better learning of both word order and case marking compared to their L1 English counterparts. This suggests that the role of L1 transfer should be carefully considered when investigating incidental learning of novel grammatical structures. Finally, the study revealed a strong link between metalinguistic awareness and language learning rates, highlighting, once again, the role of awareness in L2 learning. Importantly, however, participants exhibited some signs of learning during the initial stages of exposure and before there was an observable effect of metalinguistic awareness, bringing up the question of how explicit knowledge arises in incidental contexts of exposure. Earlier work from the field of consciousness suggests that explicit knowledge may emerge from implicit representations [170–172]. Further research is needed to shed light on this topic.

Furthermore, it should be acknowledged that the learning outcomes of both language groups are characterized by large individual differences. While examining the sources of individual variation was outside the focus of the current study, future research will benefit from investigating how individual differences relate to the learning of various grammatical structures under incidental exposure conditions. In addition, although grammar learning was found to be modulated by L1 experience, this effect emerged only after participants had received adequate input. It is, hence, recommended that future work consider including more than a single session in order to better gauge any potential L1 effects. Whether further increasing the typological similarity between the artificial language and participants’ L1 (e.g., Russian or Japanese, which mark case on the noun rather than the determiner, as in German) would facilitate learning also remains to be seen.

## Supporting information

S1 Appendix Complete list of pseudowords used in the experiment. (DOCX)

S2 Appendix (DOCX)

S3 Appendix (DOCX)

S4 Appendix (DOCX)
