# Supplementary material for: Can adults learn L2 grammar after prolonged exposure under incidental conditions?
Source: PLoS One. 2023 Jul 26;18(7):e0288989. doi: 10.1371/journal.pone.0288989 (PMC10370733; doi:10.1371/journal.pone.0288989)
Supplement: S1 Appendix — (DOCX) [file pone.0288989.s001.docx]

**S1 Appendix.** Complete list of pseudowords used in the experiment.

|  | Nominative | Accusative |
| --- | --- | --- |
| **Nouns** |  |  |
| Alg | -i | -o |
| Prad | -i | -o |
| Torg | -i | -o |
| Urg | -i | -o |
| Flub | -a | -o |
| Ird | -a | -o |
| Olb | -a | -o |
| Velg | -a | -o |
| **Adjectives** |  |  |
| kov | -i or -a | -o |
| pog | -i or -a | -o |
| **Verbs** |  |  |
| birek |  |  |
| dolek |  |  |
| mulek |  |  |
| varek |  |  |
