# Supplementary material for: Can adults learn L2 grammar after prolonged exposure under incidental conditions?
Source: PLoS One. 2023 Jul 26;18(7):e0288989. doi: 10.1371/journal.pone.0288989 (PMC10370733; doi:10.1371/journal.pone.0288989)
Supplement: S2 Appendix — (DOCX) [file pone.0288989.s002.docx]

**Table 1.** Correlations between variables: Session 1 (L1 English learners)

|  | Lexical Training (%) | Grammatical Comprehension (%) | GJT (%) | FGT (%) | Pre-training (Acc) |
| --- | --- | --- | --- | --- | --- |
| Lexical Training (%) | 1 | .03 | .33 | .29 | .46 |
| Grammatical Comprehension (%) | .03 | 1 | .04 | -.25 | .17 |
| GJT (%) | .33 | .04 | 1 | .23 | .07 |
| FGT (%) | .29 | -.25 | .23 | 1 | -.12 |
| Pre-training (Acc) | .46 | .17 | .07 | -.12 | 1 |

**Table 2.** Correlations between variables: Session 2 (L1 English learners)

|  | Lexical Training (%) | Grammatical Comprehension (%) | GJT (%) | FGT (%) | Pre-training (Acc) |
| --- | --- | --- | --- | --- | --- |
| Lexical Training (%) | 1 | .25 | .46 | .17 | .51 |
| Grammatical Comprehension (%) | .25 | 1 | .19 | -.09 | .10 |
| GJT (%) | .46 | .19 | 1 | .23 | .07 |
| FGT (%) | .17 | -.09 | .23 | 1 | -.12 |
| Pre-training (Acc) | .51 | .10 | .07 | -.12 | 1 |

**Table 3.** Correlations between variables: Session 3 (L1 English learners)

|  | Lexical Training (%) | Grammatical Comprehension (%) | GJT (%) | FGT (%) | Pre-training (Acc) |
| --- | --- | --- | --- | --- | --- |
| Lexical Training (%) | 1 | .16 | .39 | .17 | .48 |
| Grammatical Comprehension (%) | .16 | 1 | .28 | -.02 | .08 |
| GJT (%) | .39 | .28 | 1 | .23 | .07 |
| FGT (%) | .17 | -.02 | .23 | .1 | -.12 |
| Pre-training (Acc) | .48 | .08 | .07 | -.12 | 1 |

**Table 4.** Correlations between variables: Session 4 (L1 English learners)

|  | Lexical Training (%) | Grammatical Comprehension (%) | GJT (%) | FGT (%) | Pre-training (Acc) |
| --- | --- | --- | --- | --- | --- |
| Lexical Training (%) | 1 | .28 | .39 | .18 | .42 |
| Grammatical Comprehension (%) | .28 | 1 | .40 | .10 | .07 |
| GJT (%) | .39 | .40 | 1 | .23 | .07 |
| FGT (%) | .18 | .10 | .23 | .1 | -.12 |
| Pre-training (Acc) | .42 | .07 | .07 | -.12 | 1 |

**Table 5.** Correlations between variables: Session 1 (L1 German learners)

|  | _Lexical Training (%)_ | _Grammatical Comprehension (%)_ | _GJT1 (%)_ | _GJT2 (%)_ | _FGT1 (%)_ | _FGT2 (%)_ | _Pre-training (Acc)_ | _Metalinguistic awareness (Acc)_ |
| --- | --- | --- | --- | --- | --- | --- | --- | --- |
| _Lexical Training (%)_ | _1_ | _.31_ | _.53_ | _.37_ | _.25_ | _.28_ | _.59_ | _.52_ |
| _Grammatical Comprehension (%)_ | _.31_ | _1_ | _.24_ | _.04_ | _.15_ | _.04_ | _.01_ | _.06_ |
| _GJT1 (%)_ | _.53_ | _.24_ | _1_ | _.71_ | _.57_ | _.52_ | _.22_ | _.74_ |
| _GJT2 (%)_ | _.37_ | _.04_ | _.71_ | _1_ | _.38_ | _.36_ | _.23_ | _.71_ |
| _FGT1 (%)_ | _.25_ | _.15_ | _.57_ | _.38_ | _1_ | _.75_ | _.13_ | _.39_ |
| _FGT2 (%)_ | _.28_ | _.04_ | _.52_ | _.36_ | _.75_ | _1_ | _.24_ | _.53_ |
| _Pre-training (Acc)_ | _.59_ | _.01_ | _.22_ | _.23_ | _.13_ | _.24_ | _1_ | _.34_ |
| _Metalinguistic Metalinguistic awareness (Acc)_ | _.52_ | _.06_ | _.74_ | _.71_ | _.39_ | _.53_ | _.34_ | _1_ |

**Table 6.** Correlations between variables: Session 2 (L1 German learners)

|  | _Lexical Training (%)_ | _Grammatical Comprehension (%)_ | _GJT1 (%)_ | _GJT2 (%)_ | _FGT1 (%)_ | _FGT2 (%)_ | _Pre-training (Acc)_ | _Metalinguistic awareness (Acc)_ |
| --- | --- | --- | --- | --- | --- | --- | --- | --- |
| _Lexical Training (%)_ | _1_ | _.45_ | _.52_ | _.48_ | _.26_ | _.26_ | _.65_ | _.50_ |
| _Grammatical Comprehension (%)_ | _.45_ | _1_ | _.54_ | _.31_ | _.46_ | _.40_ | _.30_ | _.45_ |
| _GJT1 (%)_ | _.52_ | _.54_ | _1_ | _.71_ | _.57_ | _.52_ | _.22_ | _.74_ |
| _GJT2 (%)_ | _.48_ | _.31_ | _.71_ | _1_ | _.38_ | _.36_ | _.23_ | _.71_ |
| _FGT1 (%)_ | _.26_ | _.46_ | _.57_ | _.38_ | _1_ | _.75_ | _.13_ | _.39_ |
| _FGT2 (%)_ | _.26_ | _.40_ | _.52_ | _.36_ | _.75_ | _1_ | _.24_ | _.53_ |
| _Pre-training (Acc)_ | _.65_ | _.30_ | _.22_ | _.23_ | _.13_ | _.24_ | _1_ | _.34_ |
| _Metalinguistic awareness (Acc)_ | _.50_ | _.45_ | _.74_ | _.71_ | _.39_ | _.53_ | _.34_ | _1_ |

**Table 7.** Correlations between variables: Session 3 (L1 German learners)

|  | _Lexical Training (%)_ | _Grammatical Comprehension (%)_ | _GJT1 (%)_ | _GJT2 (%)_ | _FGT1 (%)_ | _FGT2 (%)_ | _Pre-training (Acc)_ | _Metalinguistic awareness (Acc)_ |
| --- | --- | --- | --- | --- | --- | --- | --- | --- |
| _Lexical Training (%)_ | _1_ | _.56_ | _.58_ | _.54_ | _.29_ | _.33_ | _.58_ | _.56_ |
| _Grammatical Comprehension (%)_ | _.56_ | _1_ | _.70_ | _.53_ | _.62_ | _.56_ | _.19_ | _.57_ |
| _GJT1 (%)_ | _.58_ | _.70_ | _1_ | _.71_ | _.57_ | _.52_ | _.22_ | _.74_ |
| _GJT2 (%)_ | _.54_ | _.53_ | _.71_ | _1_ | _.38_ | _.36_ | _.23_ | _.71_ |
| _FGT1 (%)_ | _.29_ | _.62_ | _.57_ | _.38_ | _1_ | _.75_ | _.13_ | _.39_ |
| _FGT2 (%)_ | _.33_ | _.56_ | _.52_ | _.36_ | _.75_ | _1_ | _.24_ | _.53_ |
| _Pre-training (Acc)_ | _.58_ | _.19_ | _.22_ | _.23_ | _.13_ | _.24_ | _1_ | _.34_ |
| _Metalinguistic awareness (Acc)_ | _.56_ | _.57_ | _.74_ | _.71_ | _.39_ | _.53_ | _.34_ | _1_ |

**Table 8.** Correlations between variables: Session 4 (L1 German learners)

|  | _Lexical Training (%)_ | _Grammatical Comprehension (%)_ | _GJT1 (%)_ | _GJT2 (%)_ | _FGT1 (%)_ | _FGT2 (%)_ | _Pre-training (Acc)_ | _Metalinguistic awareness (Acc)_ |
| --- | --- | --- | --- | --- | --- | --- | --- | --- |
| _Lexical Training (%)_ | _1_ | _.51_ | _.65_ | _.66_ | _.28_ | _.30_ | _.48_ | _.60_ |
| _Grammatical Comprehension (%)_ | _.51_ | _1_ | _.62_ | _.59_ | _.54_ | _.50_ | _.14_ | _.53_ |
| _GJT1 (%)_ | _.65_ | _.62_ | _1_ | _.71_ | _.57_ | _.52_ | _.22_ | _.74_ |
| _GJT2 (%)_ | _.66_ | _.59_ | _.71_ | _1_ | _.38_ | _.36_ | _.23_ | _.71_ |
| _FGT1 (%)_ | _.28_ | _.54_ | _.57_ | _.38_ | _1_ | _.75_ | _.13_ | _.39_ |
| _FGT2 (%)_ | _.30_ | _.50_ | _.52_ | _.36_ | _.75_ | _1_ | _.24_ | _.53_ |
| _Pre-training (Acc)_ | _.48_ | _.14_ | _.22_ | _.23_ | _.13_ | _.24_ | _1_ | _.34_ |
| _Metalinguistic awareness (Acc)_ | _.60_ | _.53_ | _.74_ | _.71_ | _.39_ | _.53_ | _.34_ | _1_ |

**Table 9.** Correlations between variables: Session 5 (L1 German learners)

|  | _Lexical Training (%)_ | _Grammatical Comprehension (%)_ | _GJT1 (%)_ | _GJT2 (%)_ | _FGT1 (%)_ | _FGT2 (%)_ | _Pre-training (Acc)_ | _Metalinguistic awareness (Acc)_ |
| --- | --- | --- | --- | --- | --- | --- | --- | --- |
| _Lexical Training (%)_ | _1_ | _.65_ | _.64_ | _.62_ | _.28_ | _.34_ | _.45_ | _.64_ |
| _Grammatical Comprehension (%)_ | _.65_ | _1_ | _.59_ | _.55_ | _.50_ | _.53_ | _.21_ | _.63_ |
| _GJT1 (%)_ | _.64_ | _.59_ | _1_ | _.71_ | _.57_ | _.52_ | _.22_ | _.74_ |
| _GJT2 (%)_ | _.62_ | _.55_ | _.71_ | _1_ | _.38_ | _.36_ | _.23_ | _.71_ |
| _FGT1 (%)_ | _.28_ | _.50_ | _.57_ | _.38_ | _1_ | _.75_ | _.13_ | _.39_ |
| _FGT2 (%)_ | _.34_ | _.53_ | _.52_ | _.36_ | _.75_ | _1_ | _.24_ | _.53_ |
| _Pre-training (Acc)_ | _.45_ | _.21_ | _.22_ | _.23_ | _.13_ | _.24_ | _1_ | _.34_ |
| _Metalinguistic awareness (Acc)_ | _.64_ | _.63_ | _.74_ | _.71_ | _.39_ | _.53_ | _.34_ | _1_ |

**Table 10.** Correlations between variables: Session 6 (L1 German learners)

|  | _Lexical Training (%)_ | _Grammatical Comprehension (%)_ | _GJT1 (%)_ | _GJT2 (%)_ | _FGT1 (%)_ | _FGT2 (%)_ | _Pre-training (Acc)_ | _Metalinguistic awareness (Acc)_ |
| --- | --- | --- | --- | --- | --- | --- | --- | --- |
| _Lexical Training (%)_ | _1_ | _.59_ | _.56_ | _.61_ | _.33_ | _.38_ | _.49_ | _.62_ |
| _Grammatical Comprehension (%)_ | _.59_ | _1_ | _.53_ | _.56_ | _.47_ | _.50_ | _.22_ | _.62_ |
| _GJT1 (%)_ | _.56_ | _.53_ | _1_ | _.71_ | _.57_ | _.52_ | _.22_ | _.74_ |
| _GJT2 (%)_ | _.61_ | _.56_ | _.71_ | _1_ | _.38_ | _.36_ | _.23_ | _.71_ |
| _FGT1 (%)_ | _.33_ | _.47_ | _.57_ | _.38_ | _1_ | _.75_ | _.13_ | _.39_ |
| _FGT2 (%)_ | _.38_ | _.50_ | _.52_ | _.36_ | _.75_ | _1_ | _.24_ | _.53_ |
| _Pre-training (Acc)_ | _.49_ | _.22_ | _.22_ | _.23_ | _.13_ | _.24_ | _1_ | _.34_ |
| _Metalinguistic awareness (Acc)_ | _.62_ | _.62_ | _.74_ | _.71_ | _.39_ | _.53_ | _.34_ | _1_ |
