# Supplementary material for: Can adults learn L2 grammar after prolonged exposure under incidental conditions?
Source: PLoS One. 2023 Jul 26;18(7):e0288989. doi: 10.1371/journal.pone.0288989 (PMC10370733; doi:10.1371/journal.pone.0288989)
Supplement: S3 Appendix — (DOCX) [file pone.0288989.s003.docx]

**Table 1.** Spearman-Brown corrected reliability estimates for all test measures in Experiment 1

| Test measure | Reliability | |
| --- | --- | --- |
|  | r_SB_ | 95%CI |
| Grammatical comprehension test |  |  |
| *Session 1* | 0.84 | [0.76, 0.90] |
| *Session 2* | 0.84 | [0.76, 0.90] |
| *Session 3* | 0.80 | [0.70, 0.88] |
| *Session 4* | 0.88 | [0.81, 0.93] |
| Grammaticality Judgement Task | 0.46 | [0.19,0.67] |
| Final Grammatical Comprehension test | 0.44 | [0.16, 0.66] |

Note: Reliability estimates for the Grammaticality Judgement Task and the Final Grammatical Comprehension test were much higher when looking separately at the different types of sentences (Grammaticality Judgement Task: *r_SB_* = 0.89 [0.81, 0.95] for grammatical sentences; *r_SB_* = 0.74 [0.61, 0.84] for ungrammatical sentences; Final Grammatical Comprehension test: *r_SB_* = 0.84 [0.75, 0.90] for OSV sentences; *r_SB_* = 0.77 [0.65, 0.87] for SOV sentences).

**Table 2.** Spearman-Brown corrected reliability estimates for all test measures in Experiment 2

| Test measure | Reliability | |
| --- | --- | --- |
|  | r_SB_ | 95%CI |
| Grammatical comprehension test |  |  |
| *Session 1* | 0.91 | [0.86, 0.95] |
| *Session 2* | 0.91 | [0.86, 0.95] |
| *Session 3* | 0.92 | [0.88, 0.96] |
| *Session 4* | 0.94 | [0.91, 0.97] |
| *Session 5* | 0.93 | [0.89, 0.96] |
| *Session 6* | 0.94 | [0.90, 0.97] |
| Grammaticality Judgement Task 1 | 0.84 | [0.75, 0.91] |
| Grammaticality Judgement Task 2 | 0.87 | [0.79, 0.92] |
| Final Grammatical Comprehension test 1 | 0.89 | [0.83, 0.94] |
| Final Grammatical Comprehension test 2 | 0.92 | [0.87, 0.95] |
