# Supplementary material for: Can adults learn L2 grammar after prolonged exposure under incidental conditions?
Source: PLoS One. 2023 Jul 26;18(7):e0288989. doi: 10.1371/journal.pone.0288989 (PMC10370733; doi:10.1371/journal.pone.0288989)
Supplement: S4 Appendix — (DOCX) [file pone.0288989.s004.docx]

**Table 1**. Mixed-effects model fitted to the Grammatical Judgement Task data.

| Variable | *B* | *SE* | *Z* | *P* | *Odds Ratios (CI)* |
| --- | --- | --- | --- | --- | --- |
| (Intercept) | 1.799 | 0.261 | 6.899 | <.001 | 6.04 (3.62 - 10.07) |
| Word Order | 0.167 | 0.398 | 0.419 | .675 | 1.18 (0.54 - 2.58) |
| Grammaticality | 4.536 | 0.663 | 6.844 | <.001 | 93.28 (25.45 - 341.93) |
| Pretraining | 0.288 | 0.144 | 2.003 | .045 | 1.33 (1.01 - 1.77) |
| Error Type | 2.940 | 0.467 | 6.292 | <.001 | 18.91 (7.57 - 47.26) |
| Grammaticality: Word Order | 0.580 | 0.774 | 0.750 | .453 | 1.79 (0.39 - 8.14) |
| Grammaticality: Pretraining | 1.298 | 0.485 | 2.675 | .007 | 3.66 (1.41 - 9.48) |
| Word Order: Pretraining | -0.175 | 0.116 | -1.510 | .131 | 0.84 (0.67 - 1.05) |
| Word Order: Error Type | 0.416 | 0.792 | 0.525 | . 599 | 1.52 (0.32 - 7.15) |
| Grammaticality: Error Type | -4.466 | 0.817 | -5.465 | <.001 | 0.01 (0.00 - 0.06) |
| Pretraining: Error Type | 0.438 | 0.211 | 2.075 | .038 | 1.55 (1.02 - 2.34) |
| *Random effects* | Variance | SD |  |  |  |
| Item (intercept) | 2.440 | 1.562 |  |  |  |
| Participant (intercept) | 0.620 | 0.787 |  |  |  |
| Participant \| Grammaticality (slope) | 8.521 | 2.919 |  |  |  |
| Participant \| Error Type (slope) | 1.065 | 1.032 |  |  |  |
| Marginal R^2^ | .537 |  |  |  |  |
| Conditional R^2^ | .824 |  |  |  |  |

**Table 2**. Mixed-effects model fitted to the Final Grammatical Comprehension test data.

| Variable | *B* | *SE* | *Z* | *P* | | *Odds Ratios (CI)* |
| --- | --- | --- | --- | --- | --- | --- |
| (Intercept) | 0.194 | 0.144 | 1.345 | .179 | | 1.21 (0.92 - 1.61) |
| Word Order | 2.522 | 0.455 | 5.538 | <.001 | | 12.45 (5.10 - 30.40) |
| Pretraining | 0.035 | 0.095 | 0.374 | .708 | | 1.04 (0.86 - 1.25) |
| Word Order: Pretraining | 0.055 | 0.399 | 0.137 | .891 | | 1.06 (0.48 - 2.31) |
| *Random effects* | Variance | SD |  |  |  | |
| Item (intercept) | 0.217 | 0.466 |  |  |  | |
| Participant (intercept) | 5.871 | 2.423 |  |  |  | |
| Participant \| Word Order (slope) | 0.411 | 0.641 |  |  |  | |
| Marginal R^2^ | .228 |  |  |  |  | |
| Conditional R^2^ | .529 |  |  |  |  | |

**Table 3**. Mixed-effects model fitted to the Grammatical Judgement Task data from L1 German participants.

| Variable | *B* | *SE* | *Z* | *P* | *Odds Ratios (CI)* | |
| --- | --- | --- | --- | --- | --- | --- |
| (Intercept) | 2.384 | 0.198 | 12.05 | <.001 | 10.85 (7.36 - 15.99) | |
| Word Order | 0.236 | 0. 272 | 0.870 | .384 | 1.27 (0.74 - 2.16) | |
| Grammaticality | 3.226 | 0.482 | 6.695 | <.001 | 25.19 (9.79 - 64.77) | |
| Pretraining | 0.244 | 0.145 | 1.685 | .092 | 1.28 (0.96 - 1.69) | |
| Test Time | 0.345 | 0.272 | 1.268 | .205 | 1.41 (0.83 - 2.41) | |
| Metalinguistic Awareness | 1.018 | 0.151 | 6.725 | <.001 | 2.77 (2.06 - 3.72) | |
| Error Type | 2.206 | 0.297 | 7.428 | <.001 | 9.08 (5.07 - 16.24) | |
| Word Order: Grammaticality | 0.788 | 0.532 | 1.479 | .139 | 2.20 (0.77 - 6.24) | |
| Word Order: Pretraining | -0.154 | 0.110 | -1.407 | .159 | 0.86 (0.69 - 1.06) | |
| Word Order: Test Time | -0.015 | 0.523 | -0.030 | .976 | 0.98 (0.35 - 2.74) | |
| Word Order: Metalinguistic Awareness | -0.070 | 0.136 | -0.514 | .607 | 0.93 (0.72 - 1.22) | |
| Word Order: Error Type | -0.138 | 0.530 | -0.260 | .795 | 0.87 (0.31 - 2.46) | |
| Grammaticality: Pretraining | 0.878 | 0.426 | 2.062 | .039 | 2.41 (1.04 - 5.55) | |
| Grammaticality: Test Time | -0.283 | 0.540 | -0.525 | .60 | 0.75 (0.26 - 2.17) | |
| Grammaticality: Metalinguistic Awareness | -1.507 | 0.424 | -3.551 | <.001 | 0.22 (0.10 - 0.51) | |
| Grammaticality: Error Type | -5.020 | 0.572 | -8.783 | <.001 | 0.01 (0.00 - 0.02) | |
| Pretraining: Test Time | 0.075 | 0.109 | 0.686 | .493 | 1.08 (0.87 - 1.34) | |
| Pretraining: Metalinguistic Awareness | 0.225 | 0.160 | 1.401 | .161 | 1.25 (0.91 - 1.71) | |
| Pretraining: Error Type | 0.400 | 0.154 | 2.600 | .009 | 1.49 (1.10 - 2.02) | |
| Test Time: Metalinguistic Awareness | 0.250 | 0.125 | 2.002 | .045 | 1.28 (1.01 - 1.64) | |
| Test Time: Error Type | 0.566 | 0.538 | 1.052 | .293 | 1.76 (0.61 - 5.06) | |
| Metalinguistic Awareness: Error Type | -0.215 | 0.181 | -1.191 | .234 | 0.81 (0.57 - 1.15) | |
| *Random effects* | Variance | SD |  |  | |  |
| Item (intercept) | 1.156 | 1.075 |  |  | |  |
| Participant (intercept) | 0.438 | 0.662 |  |  | |  |
| Item \| Test Time (slope) | 3.899 | 1.975 |  |  | |  |
| Item \| Metalinguistic Awareness (slope) | 0.168 | 0.410 |  |  | |  |
| Participant \| Grammaticality (slope) | 5.201 | 2.281 |  |  | |  |
| Participant \| Error Type (slope) | 0.217 | 0.465 |  |  | |  |
| Marginal R^2^ | .524 |  |  |  | |  |
| Conditional R^2^ | .788 |  |  |  | |  |

**Table 4**. Mixed-effects model fitted to the Final Grammatical Comprehension test data from L1 German participants.

| Variable | *B* | *SE* | *Z* | *P* | *Odds Ratios (CI)* |
| --- | --- | --- | --- | --- | --- |
| (Intercept) | 1.250 | 0.248 | 5.035 | <.001 | 3.49 (2.15 - 5.68) |
| Word Order | 2.865 | 0.552 | 5.192 | <.001 | 17.54 (5.95 - 51.72) |
| Pretraining | 0.036 | 0.248 | 0.145 | .885 | 1.04 (0.64 - 1.69) |
| Test Time | 0.377 | 0.139 | 2.715 | .007 | 1.46 (1.11 - 1.92) |
| Metalinguistic Awareness | 1.960 | 0.270 | 7.262 | <.001 | 7.10 (4.18 - 12.05) |
| Word Order: Pretraining | 0.474 | 0.553 | 0.857 | .391 | 1.61 (0.54 - 4.75) |
| Word Order: Test Time | 0.280 | 0.255 | 1.097 | .273 | 1.32 (0.80 - 2.18) |
| Word Order: Metalinguistic Awareness | -0.998 | 0.619 | -1.614 | .107 | 0.37 (0.11 - 1.24) |
| Pretraining: Test Time | 0.051 | 0.131 | 0.393 | .694 | 1.05 (0.81 - 1.36) |
| Pretraining: Metalinguistic Awareness | 0.558 | 0.293 | 1.904 | .057 | 1.75 (0.98 - 3.10) |
| Test Time: Metalinguistic Awareness | 0.359 | 0.191 | 1.882 | .060 | 1.43 (0.99 - 2.08) |
| *Random effects* | Variance | SD |  |  |  |
| Item (intercept) | 0.141 | 0.375 |  |  |  |
| Participant (intercept) | 1.337 | 1.156 |  |  |  |
| Participant \| Word Order (slope) | 8.532 | 2.921 |  |  |  |
| Marginal R^2^ | .480 |  |  |  |  |
| Conditional R^2^ | .752 |  |  |  |  |

**Table 5**. Mixed-effects model fitted to the first Grammatical Judgement Task data with both L1 groups.

| Variable | *B* | *SE* | *Z* | *P* | *Odds Ratios (CI)* |
| --- | --- | --- | --- | --- | --- |
| (Intercept) | 1.948 | 0.205 | 9.504 | <.001 | 7.02 (4.70 - 10.49) |
| Grammaticality | 3.731 | 0.478 | 7.806 | <.001 | 41.71 (16.35 - 106.44) |
| Group | 0.402 | 0.251 | 1.600 | .110 | 1.49 (0.91 - 2.45) |
| Error Type | 2.395 | 0.363 | 6.603 | <.001 | 10.97 (5.39 - 22.34) |
| Grammaticality: Group | -1.156 | 0.694 | -1.666 | .096 | 0.31 (0.08 - 1.23) |
| Grammaticality: Error Type | -4.159 | 0.679 | -6.124 | <.001 | 0.02 (0.00 - 0.06) |
| Group: Error Type | -0.792 | 0.339 | -2.339 | .019 | 0.45 (0.23 - 0.88) |
| Grammaticality: Group: Error Type | 0.842 | 0.478 | 1.762 | .078 | 2.32 (0.91 - 5.92) |
| *Random effects* | Variance | SD |  |  |  |
| Item (intercept) | 1.900 | 1.378 |  |  |  |
| Participant (intercept) | 0.855 | 0.924 |  |  |  |
| Item \| Group (slope) | 0.225 | 0.475 |  |  |  |
| Participant \| Grammaticality (slope) | 7.996 | 2.828 |  |  |  |
| Participant \| Error Type (slope) | 0.927 | 0.963 |  |  |  |
| Marginal R^2^ | .457 |  |  |  |  |
| Conditional R^2^ | .785 |  |  |  |  |

**Table 7**. Mixed-effects model fitted to the first Final Grammatical Comprehension test data with both L1 groups.

| Variable | *B* | *SE* | *Z* | *P* | *Odds Ratios (CI)* |
| --- | --- | --- | --- | --- | --- |
| (Intercept) | 0.619 | 0.179 | 3.458 | <.001 | 1.86 (1.31 - 2.64) |
| Group | 0.804 | 0.297 | 2.708 | .007 | 2.23 (1.25 - 4.00) |
| Word Order | 2.635 | 0.385 | 6.839 | <.001 | 13.94 (6.55 - 29.66) |
| Word Order: Group | 0.051 | 0. 659 | 0.077 | .938 | 1.05 (0.29 - 3.83) |
| *Random effects* | Variance | SD |  |  |  |
| Item (intercept) | 1.456 | 1.206 |  |  |  |
| Participant (intercept) | 0.366 | 0.605 |  |  |  |
| Participant\| Word Order (slope) | 7.435 | 2.727 |  |  |  |
| Marginal R^2^ | .214 |  |  |  |  |
| Conditional R^2^ | .629 |  |  |  |  |
